# Identification and characterization of *ethR* and *ethA* genes impacting the sensitivity of *Mycobacterium abscessus* to ethionamide

**DOI:** 10.1128/spectrum.00638-26

**Published:** 2026-08-28

**Authors:** Xiaofan Zhang, Lijie Li, Ziwen Lu, Chaohui Duan, H. M. Adnan Hameed, Aweke Mulu Belachew, Cuiting Fang, Xirong Tian, Xinyue Wang, Hongyi Chen, Liqiang Feng, Matthew B. McNeil, Dongxin Liu, Shuai Wang, Tianyu Zhang

**Affiliations:** 1 State Key Laboratory of Respiratory Disease, Guangzhou Institutes of Biomedicine and Health, Chinese Academy of Sciences 74627, Guangzhou, China; 2 University of Chinese Academy of Sciences 74519, Beijing, China; 3 Department of Clinical Laboratory, Sun Yat-sen Memorial Hospital of Sun Yat-sen University 162697, Guangzhou, China; 4 Guangdong-Hong Kong-Macao Joint Laboratory of Respiratory Infectious Diseases, Guangzhou Institutes of Biomedicine and Health, Chinese Academy of Sciences 74627, Guangzhou, China; 5 School of Life Sciences, University of Science and Technology of China 12652, Hefei, China; 6 Guangzhou Medical University-Guangzhou Institutes of Biomedicine and Health Joint School of Life Sciences, Guangzhou Medical University 26468, Guangzhou, China; 7 State Key Laboratory of Respiratory Disease, Guangzhou Chest Hospital 555049, Guangzhou, China; 8 Guangzhou National Laboratory 612039, Guangzhou, China; 9 West China School of Public Health, Sichuan University 12530, Chengdu, China; 10 Department of Microbiology and Immunology, School of Biomedical Sciences, University of Otago 2495, Dunedin, New Zealand; 11 Maurice Wilkins Centre for Molecular Biodiscovery, University of Auckland 1415, Auckland, New Zealand; 12 Chinese Center for Disease Control and Prevention, National Pathogen Resource Center 12415, Beijing, China; MultiCare Health System, Tacoma, Washington, USA

**Keywords:** *Mycobacterium abscessus*, ethionamide, intrinsic resistance, *ethR*, *ethA*

## Abstract

*Mycobacterium abscessus* is a rapidly growing non-tuberculous mycobacterium with rising global incidence. This pathogen exhibits intrinsic resistance to most antibiotics, presenting a major public health threat. Ethionamide (ETH) requires bioactivation by monooxygenase EthA to form the active ETH-NAD adduct. We previously identified MAB_3513 (NudC) as a phosphohydrolase that confers intrinsic resistance to *M. abscessus* by hydrolyzing this adduct. However, deletion of *nudC* results in only partial susceptibility to ETH, indicating the existence of additional resistance mechanisms. This study identified MAB_0984 as the EthR regulator in *M. abscessus*. Deletion of *ethR* in a *nudC* knockout background (ΔΔ*ethR*) significantly enhanced ETH-NAD adduct accumulation, leading to hypersusceptibility to ETH. Notably, the Δ*ethR* mutant exhibited higher susceptibility than Δ*nudC*, demonstrating that EthR is a more dominant mediator of ETH resistance than NudC. Furthermore, MAB_0985 (EthA1) and MAB_0103 (EthA2) are the principal ETH-activating monooxygenases in *M. abscessus*. Deletion of either gene alone or in combination in the Δ*nudC* reduced adduct formation and increased resistance, while the triple mutant ΔΔ*ethA1*Δ*ethA2* restored wild-type resistance. Using an intergenic region-enhanced green fluorescent protein reporter system and quantitative reverse transcription-PCR, we demonstrated that EthR confers resistance by specifically suppressing *ethA1* expression in *M. abscessus*. The *Mycobacterium tuberculosis* EthR inhibitor BDM31343 could boost the efficacy of ETH against *M. abscessus* by inhibiting EthR. Collectively, this study identified the *ethR* and *ethA* genes in *M. abscessus* for the first time and elucidated their role in mediating resistance to ETH. Therefore, EthR is a promising target for potentiating the efficacy of ETH against *M. abscessus*.

**IMPORTANCE:**

*Mycobacterium abscessus* constitutes an escalating global health threat, primarily due to its intrinsic resistance to most antibiotics. This study identifies MAB_0984 (EthR) as a dominant resistance determinant that exerts a more profound impact on ethionamide (ETH) susceptibility than the previously characterized NudC. We demonstrate that EthR mediates resistance by specifically repressing the expression of EthA1 (MAB_0985), one of the primary monooxygenases responsible for ETH bioactivation. The *Mycobacterium tuberculosis* EthR inhibitor BDM31343 potentiated the activity of ETH against *M. abscessus* through inhibition of EthR. These findings elucidate the mechanism of ETH resistance in *M. abscessus*, identifying EthR as a promising target for boosting the efficacy of ETH against *M. abscessus*.

## INTRODUCTION

*Mycobacterium abscessus* (*M. abscessus*) is a rapidly growing nontuberculous mycobacterium (NTM) that causes severe pulmonary, cutaneous, and subcutaneous infections in humans. Over the past two decades, the global incidence of NTM infections has increased markedly across diverse geographic regions, including North America, Europe, Asia, and Australia (1). In developed countries, the incidence and prevalence of NTM pulmonary disease now exceed those of tuberculosis (2). Among NTMs, *M. abscessus* is the second most common pathogen causing NTM disease and the most prevalent rapidly growing mycobacterial species, with disease burden particularly pronounced in aging populations (1). As an environmental opportunistic pathogen, *M. abscessus* is widely distributed in the environment, and recent studies have also confirmed its potential for person-to-person transmission (3). Together, these factors underscore *M. abscessus* as an emerging public health threat that demands urgent attention.

The clinical management of *M. abscessus* infections is severely compromised by its extensive intrinsic and acquired resistance to multiple antibiotics. This multidrug resistance is driven by various mechanisms, including a highly impermeable lipid-rich cell envelope, active drug efflux pumps, antibiotic-modifying enzymes, and genetic alterations affecting drug targets (4). Consequently, the extensive nature of drug resistance, combined with the lack of consistently effective treatment regimens, makes it extremely difficult to treat these infections. Treatment outcomes for *M. abscessus* infections are often poor, with combination antibiotic therapy achieving success in only 30%–50% of patients, and surgical resection is frequently required as an adjunctive intervention to control the infection (5). Current guidelines recommend prolonged treatment regimens for *M. abscessus* pulmonary disease, typically involving 6–12 months of combined oral and intravenous antibiotics (6), followed by continuation of therapy for at least 12 months after sputum culture conversion (7). Oral regimens generally consist of one or two agents, predominantly including macrolides, linezolid, or clofazimine, whereas fluoroquinolones are occasionally incorporated (6). Intravenous therapy commonly involves two or more drugs, including amikacin (AMK), tigecycline, imipenem, or cefoxitin (6). Although newer agents such as bedaquiline (BDQ) and its next-generation derivatives TBAJ-587 and TBAJ-876 have demonstrated promising *in vitro* and *in vivo* activity against *M. abscessus* (8, 9), treatment success remains suboptimal, particularly in chronic, refractory pulmonary disease, which is frequently associated with relapse and increased mortality. These challenges highlight an urgent need to elucidate the molecular basis of antibiotic resistance in *M. abscessus* and identify novel therapeutic targets.

Ethionamide (ETH) is a second-line antitubercular drug widely used for the treatment of multidrug-resistant tuberculosis. As a prodrug and structural analog of isoniazid (INH), ETH is activated by the bacterial flavin-dependent monooxygenases to form an active ETH-NAD adduct. This adduct inhibits the enoyl-acyl carrier protein reductase (InhA) in the fatty acid synthase II pathway, disrupting mycolic acid synthesis and ultimately causing mycobacterial cell death (10). Three monooxygenases identified in *Mycobacterium tuberculosis*, EthA^Mtb^ (Rv3854) (11), EthA2^Mtb^ (Rv0077) (12), and MymA (Rv3083) (13), have been shown to activate ETH. The expression of these enzymes is tightly regulated by the transcriptional regulators EthR^Mtb^ (Rv3855) (11), EthR2^Mtb^ (Rv0078) (12), and VirS (Rv3082) (13), respectively. EthR^Mtb^ and EthR2^Mtb^ are members of the TetR family of transcriptional repressors, whereas VirS belongs to the AraC/XylS family of transcriptional regulators. Overexpression of *ethA*^Mtb^, *ethA2*^Mtb^, or *mymA* increased *M. tuberculosis* susceptibility to ETH (11–13). Furthermore, small-molecule inhibitors targeting these transcriptional regulators, BDM41906, SMARt420, and SMARt751, have been shown to derepress monooxygenase expression, thereby markedly potentiating ETH activity both *in vitro* and *in vivo* (12, 14, 15). In contrast, *M. abscessus* exhibits high-level intrinsic resistance to ETH, and the precise molecular mechanism underlying this resistance remains incompletely characterized. Our prior investigation demonstrated that the *MAB_3513c* gene in *M. abscessus* encodes NudC, a pyrophosphohydrolase enzyme that confers ETH resistance by hydrolyzing the ETH-NAD adduct (16). Although the *nudC* knockout reduced the minimal inhibitory concentration (MIC) of ETH against *M. abscessus* from 256 to 16 μg/mL, the value remains much higher than the MIC observed for *M. tuberculosis*, indicating additional resistance mechanisms in *M. abscessus*.

This study is the first to identify MAB_0984 as the functional EthR homolog in *M. abscessus*, which regulates EthA1 (MAB_0985) expression and thereby suppresses ETH-NAD adduct formation. Our findings reveal that impaired EthR-regulated activation is a more critical determinant of intrinsic ETH resistance in *M. abscessus* than the previously identified NudC-mediated detoxification pathway. Additionally, our study demonstrates that MAB_0985 and MAB_0103 function as the principal EthAs in *M. abscessus*, mediating ETH activation and the subsequent formation of ETH-NAD adduct. Moreover, the small-molecule inhibitor BDM31343, targeting EthR in *M. tuberculosis*, enhanced the efficacy of ETH against *M. abscessus*. Collectively, our findings elucidate EthR-mediated suppression as a major mechanism of intrinsic ETH resistance in *M. abscessus* and identify EthR as a promising therapeutic target for improving ETH efficacy against this highly drug-resistant pathogen.

## RESULTS

### Deletion of *MAB_0984c* encoding a homolog of the EthR^Mtb^ increased *M. abscessus* susceptibility to ETH

MAB_0984 was identified as the EthR homolog in *M. abscessus*, exhibiting 50.23% amino acid sequence identity with EthR^Mtb^ (Fig. S1). Both proteins belong to the TetR family of transcriptional regulators. In contrast, no homologs exhibiting >40% amino acid sequence identity to EthR2^Mtb^ or VirS were detected in *M. abscessus*, suggesting that MAB_0984 represents the primary EthR-like regulator in *M. abscessus*.

**Fig 1:**
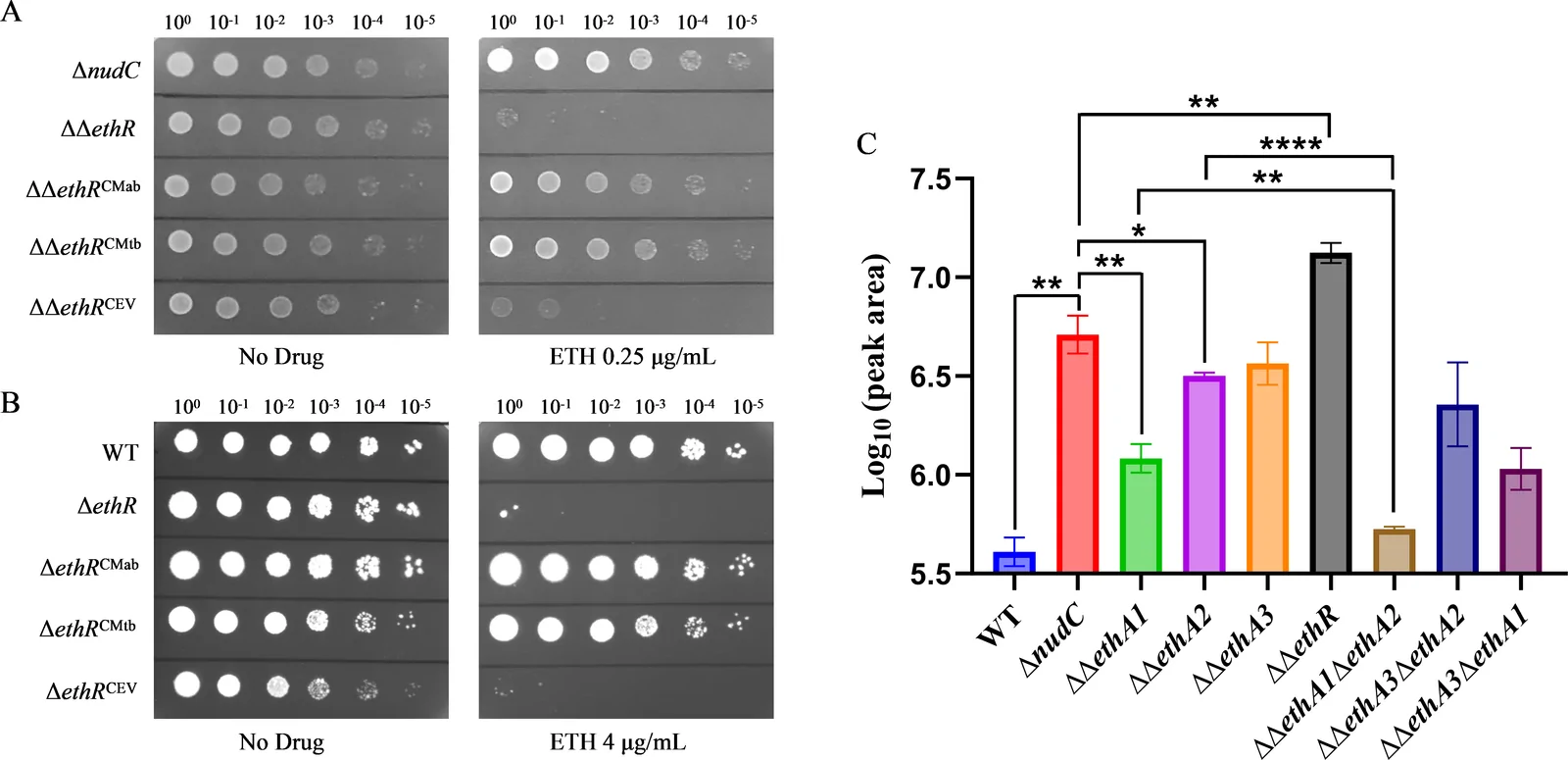
Knockout of *ethR* (*MAB_0984c*) significantly enhances susceptibility to ETH by promoting the accumulation of the ETH-NAD adduct within *M. abscessus*. (**A**) Drug susceptibility testing of the *ethR* and *nudC* double knockout strain to ETH. Δ*nudC*, *M. abscessus nudC* knockout strain; ΔΔ*ethR, ethR* and *nudC* double knockout strain; and ΔΔ*ethR*^CMab^, ΔΔ*ethR*^CMtb^, and ΔΔ*ethR*^CEV^, ΔΔ*ethR* complemented with ethR^Mab^, ethR^Mtb^, or with the empty vector, respectively. (**B**) Drug susceptibility testing of the *ethR* knockout strain to ETH. WT, wild-type *M. abscessus*; Δ*ethR*, *ethR* knockout strain; and Δ*ethR*^CMab^, Δ*ethR*^CMtb^, and Δ*ethR*^CEV^, Δ*ethR* complemented with ethR^Mab^, ethR^Mtb^, or with the empty vector, respectively. (**C**) Quantification of the ETH-NAD adduct in the various *M. abscessus* strains. ΔΔ*ethA1*, *ethA1* (*MAB_0985*) and *nudC* double knockout strain; ΔΔ*ethA2*, *ethA2* (*MAB_0103*) and *nudC* double knockout strain; ΔΔ*ethA3*, *ethA3* (*MAB_3967*) and *nudC* double knockout strain; ΔΔ*ethA1*Δ*ethA2*, *ethA1*, *ethA2*, and *nudC* triple knockout strain; ΔΔ*ethA3*Δ*ethA2*, *ethA3*, *ethA2*, and *nudC* triple knockout strain; and ΔΔ*ethA3*Δ*ethA1*, *ethA3*, *ethA1,* and *nudC* triple knockout strain. *P < 0.05, **P < 0.01, and ****P < 0.0001. Data are means ± SD of three biological replicates.

To investigate its role in ETH resistance, CRISPR-assisted non-homologous end-joining (NHEJ) technology (17) was used to knock out *MAB_0984c* (*ethR*) in Δ*nudC*. A frameshift mutation was generated near the 5′ end of *ethR* by introducing a 4-bp deletion (nucleotides 191–194), which was confirmed by Sanger sequencing (Fig. S2A and B). The resulting double mutant, Δ*nudC*Δ*ethR* (hereafter referred to as ΔΔ*ethR*), exhibited extreme sensitivity to ETH, with an MIC of 0.125 μg/mL (Table 1). This represented a 128-fold reduction relative to the MIC for Δ*nudC* (16 μg/mL) and a 2,048-fold reduction relative to the MIC for the WT strain (256 μg/mL). In contrast, their MICs to rifabutin (RFB), moxifloxacin (MXF), INH, AMK, and BDQ remained unchanged (Table 1). This finding suggests that EthR constitutes an additional critical factor, beyond NudC, conferring ETH resistance in *M. abscessus*. To further investigate this, we engineered an *ethR* knockout (Δ*ethR*) strain in WT through an in-frame deletion of 240 bp beginning at nucleotide position 46 of the coding sequence, which was verified by Sanger sequencing (Fig. S2C and D). The MIC of ETH to Δ*ethR* was 4 μg/mL, representing a 64-fold reduction compared to the WT and a 4-fold reduction relative to Δ*nudC* (Table 1). The ETH resistance was successfully restored in both ΔΔ*ethR* and Δ*ethR* upon complementation with *ethR* or *ethR*^Mtb^ (Table 1), confirming their functional homology. On agar plates, ΔΔ*ethR* and Δ*ethR* exhibited sensitivity to ETH at concentrations of 0.25 and 4 μg/mL (Fig. 1A and B), respectively, consistent with the broth microdilution results. High-performance liquid chromatography-mass spectrometry (HPLC-MS) analysis further revealed substantially increased accumulation of the ETH-NAD adduct in ΔΔ*ethR* following exposure to 256 μg/mL ETH, with a peak area ratio approximately 2.5-fold higher than that observed in Δ*nudC* (Fig. 1C). Knockout of *ethR* in both Δ*nudC* and WT did not alter their respective growth rates (Fig. S3). These results indicate that MAB_0984 is the functional EthR in *M. abscessus*, contributing to intrinsic ETH resistance alongside NudC. Notably, EthR-mediated resistance is more pronounced than that attributed to NudC in *M. abscessus*.

**TABLE 1:** The susceptibility of *M. abscessus ethR* knockout strains in a *nudC* deletion and wild-type background, as well as their complementary strains, to ETH and INH

*M. abscessus* strains	Antibiotics/MICs^a^ (μg/mL)					
	ETH	INH	AMK	RFB	BDQ	MXF
∆*nudC*	16	1	16	8	1	16
∆∆*ethR*	0.125	1	16	8	1	16
∆∆*ethR*^CMab^	16	1	/	/	/	/
∆∆*ethR*^CMtb^	32	1	/	/	/	/
∆∆*ethR*^CEV^	0.125	1	/	/	/	/
WT	256	>512	16	8	1	16
∆*ethR*	4	>512	16	8	1	16
∆*ethR*^CMab^	256	>512	/	/	/	/
∆*ethR*^CMtb^	256	>512	/	/	/	/
∆*ethR*^CEV^	4	>512	/	/	/	/

^a^ Broth microdilution method was used to determine the MICs. Δ*nudC*,* M. abscessus nudC* knockout strain; ΔΔ*ethR*,* ethR* and *nudC* double knockout strain; ΔΔ*ethR*^CMab^, ΔΔ*ethR*^CMtb^, and ΔΔ*ethR*^CEV^, ΔΔ*ethR* complemented with *ethR*^Mab^, *ethR*^Mtb^, or with the empty vector, respectively. WT, wild-type *M. abscessus*; Δ*ethR*,* ethR* knockout strain; and Δ*ethR*^CMab^, Δ*ethR*^CMtb^, and Δ*ethR*^CEV^, Δ*ethR* complemented with *ethR*^Mab^, *ethR*^Mtb^, or with the empty vector, respectively. /, no data available.

### Deletion of *MAB_0985* and *MAB_0103* encoding homologs of the EthA^Mtb^ enhanced *M. abscessus* resistance to ETH

**Fig 2:**
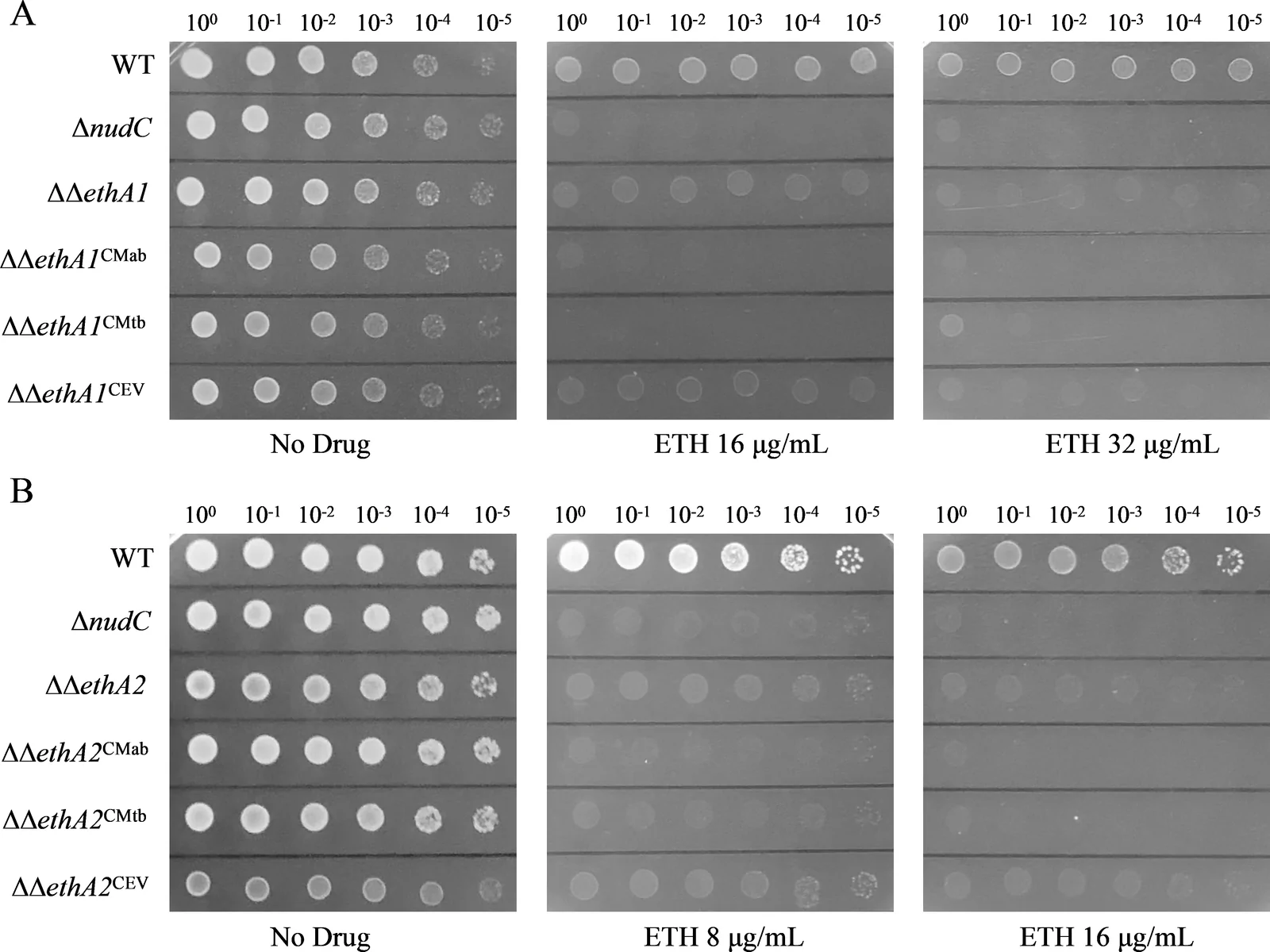
Knockout of *ethA1* (*MAB_0985*) or *ethA2* (*MAB_0103*) in the *nudC* knockout strain increases resistance to ETH. (**A**) Drug susceptibility testing of the *ethA1* and *nudC* double knockout strain to ETH. WT, wild-type *M. abscessus*; Δ*nudC, M. abscessus nudC* knockout strain; ΔΔ*ethA1, ethA1* and *nudC* double knockout strain; ΔΔ*ethA1*^CMab^, ΔΔ*ethA1*^CMtb^, and ΔΔ*ethA1*^CEV^, ΔΔ*ethA1* complemented with *ethA1*^Mab^, *ethA*^Mtb^, or with the empty vector, respectively. (**B**) Drug susceptibility testing of the *ethA2* and *nudC* double knockout strain to ETH. ΔΔ*ethA2*, *ethA2* and *nudC* double knockout strain; ΔΔ*ethA2*^CMab^, ΔΔ*ethA2*^CMtb^, and ΔΔ*ethA2*^CEV^, ΔΔ*ethA2* complemented with *ethA2*^Mab^, *ethA*^Mtb^, or with the empty vector, respectively.

Given that MAB_0984 functions as EthR in *M. abscessus* and that EthR acts by regulating EthA expression, a critical question arises: which gene serves as the functional EthA target in *M. abscessus*? BLASTp analysis identified MAB_0985, MAB_0103, and MAB_3967 in *M. abscessus* as potential EthA homologs. Pairwise amino acid sequence alignment using DNAMAN revealed that these proteins share 51.38%, 59.44%, and 47.46% sequence identity, respectively, with known EthA^Mtb^ (Fig. S4A and B). Accordingly, we have designated *MAB_0985*, *MAB_0103*, and *MAB_3967* as the ETH-activating genes *ethA1*, *ethA2*, and *ethA3,* respectively. Considering the intrinsic resistance of WT to ETH, we initially employed CRISPR-NHEJ to knockout three potential *ethA* genes in the Δ*nudC* (Fig. S5). The double knockout strains Δ*nudC*Δ*ethA1* (ΔΔ*ethA1*) and Δ*nudC*Δ*ethA2* (ΔΔ*ethA2*) demonstrated modestly elevated ETH resistance, with MICs increasing four- and twofold, respectively (Table 2). In contrast, their MICs for INH, AMK, RFB, BDQ, and MXF remained unchanged. ETH susceptibility was restored upon individual reintroduction of *ethA1*, *ethA2*, or *ethA*^Mtb^ into their respective mutants (Table S1). The enhanced ETH resistance in mutant strains was directly observable on solid plates, with ΔΔ*ethA1* exhibiting growth at 32 μg/mL and ΔΔ*ethA2* at 16 μg/mL, indicating modestly increased resistance (Fig. 2). The ΔΔ*ethA3* mutant showed no significant difference in growth from the Δ*nudC* strain on plates containing 4 or 8 μg/mL ETH (Fig. S6).

**TABLE 2:** The susceptibility of different *M. abscessus* strains to various drugs

*M. abscessus* strains	Antibiotics/MICs^a^ (μg/mL)					
	ETH	INH	AMK	RFB	BDQ	MXF
WT	256	>512	16	8	1	16
∆*nudC*	16	1	16	8	1	16
∆∆*ethA1*	64	1	16	8	1	16
∆∆*ethA2*	32	1	16	8	1	16
∆∆*ethA3*	16	1	16	8	1	16
∆*ethA1*	1,024	>512	/	/	/	/
∆*ethA2*	512	>512	/	/	/	/
∆*ethA3*	256	>512	/	/	/	/
∆∆*ethA1*∆*ethA2*	256	1	/	/	/	/
∆∆*ethA3*∆*ethA1*	64	1	/	/	/	/
∆∆*ethA3*∆*ethA2*	32	1	/	/	/	/
Δ*nudC*^OEV^	16	1	/	/	/	/
Δ*nudC*^O*ethA1*^	2	1	/	/	/	/
Δ*nudC*^O*ethA2*^	1	1	/	/	/	/
Δ*nudC*^O*ethA3*^	2	1	/	/	/	/
WT^OEV^	256	512	/	/	/	/
WT^O*ethA1*^	128	512	/	/	/	/
WT^O*ethA2*^	256	512	/	/	/	/
WT^O*ethA3*^	256	512	/	/	/	/
∆∆*cinA*	16	1	/	/	/	/
∆*cinA*	256	>512	/	/	/	/

^a^ Broth microdilution method was used to determine the MICs. WT, wild-type *M. abscessus*; Δ*nudC*, *M. abscessus nudC* knockout strain; ΔΔ*ethA1*,* ethA1* (*MAB_0985*) and *nudC* double knockout strain; ΔΔ*ethA2*,* ethA2* (*MAB_0103*) and *nudC* double knockout strain; ΔΔ*ethA3*,* ethA3* (*MAB_3967*) and *nudC* double knockout strain; Δ*ethA1*, Δ*ethA2*, and Δ*ethA3*,* ethA1, ethA2, *or* ethA3* knockout strains, respectively; Δ*nudC*^O*ethA1*^, Δ*nudC*^O*ethA2*^, and Δ*nudC*^O*ethA3*^, Δ*nudC* overexpressing *ethA1*, *ethA2*, or *ethA3*, respectively; WT^O*ethA1*^, WT^O*ethA2*^, and WT^O*ethA3*^, WT overexpressing *ethA1*, *ethA2*, or *ethA3*, respectively; ΔΔ*cinA*,* cinA* (*MAB_2443*) and *nudC* double knockout strain; and ∆*cinA*,* cinA* knockout strain. /, no data available.

**Fig 3:**
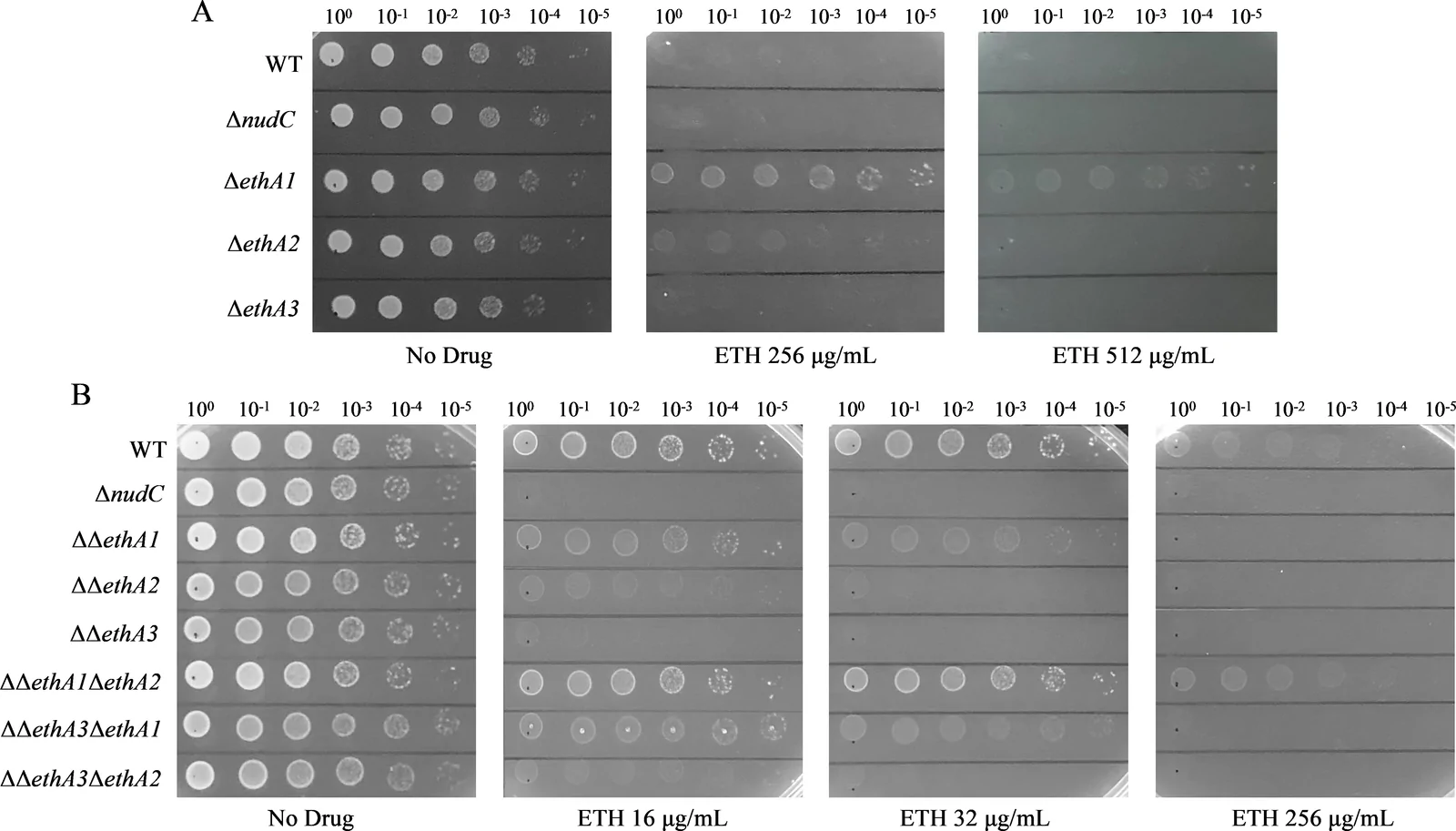
Knockout of *ethA1* (*MAB_0985*) or *ethA2* (*MAB_0103*) in wild-type *M. abscessus* increases resistance to ETH, and simultaneous knockout of *ethA1* and *ethA2* in the *nudC* knockout strain restores wild-type resistance levels. (**A**) Drug susceptibility testing of *ethA1*, *ethA2*, and *ethA3* (*MAB_3967*) knockout strain to ETH. WT, wild-type *M. abscessus*; Δ*nudC*, *M. abscessus nudC* knockout strain; Δ*ethA1*, *ethA1* knockout strain; Δ*ethA2*, *ethA2* knockout strain; and Δ*ethA3*, *ethA3* knockout strain. (**B**) Drug susceptibility testing of the triple knockout strain to ETH. ΔΔ*ethA1*, *ethA1* and *nudC* double knockout strain; ΔΔ*ethA2*, *ethA2* and *nudC* double knockout strain; ΔΔ*ethA3*, *ethA3* and *nudC* double knockout strain; ΔΔ*ethA1*Δ*ethA2*, *ethA1*, *ethA2*, and *nudC* triple knockout strain; ΔΔ*ethA3*Δ*ethA2*, *ethA3*, *ethA2*, and *nudC* triple knockout strain; and ΔΔ*ethA3*Δ*ethA1*, *ethA3*, *ethA1*, and *nudC* triple knockout strain.

To further investigate the functions of *ethA1*, *ethA2*, and *ethA3*, we generated single knockout mutants of each gene in WT (Fig. S7). Drug susceptibility testing (DST) demonstrated that Δ*ethA1* and Δ*ethA2* exhibited increased resistance to ETH compared to the WT (Table 2). The MICs to ETH rose fourfold in Δ*ethA1* and twofold in Δ*ethA2*, whereas the MIC of INH remained unchanged (Table 2). INH is specifically activated by the catalase-peroxidase KatG to form the INH-NAD adduct (10), which also subsequently inhibits InhA. Consequently, deletion of *ethA*s did not affect susceptibility to INH in the Δ*nudC*. Enhanced ETH resistance was directly demonstrated on solid medium, as evidenced by the growth of Δ*ethA1* at 512 μg/mL and Δ*ethA2* at 256 μg/mL of ETH, consistent with a resistant phenotype (Fig. 3A). The results demonstrate that deletion of *ethA1* or *ethA2* increased *M. abscessus* resistance to ETH.

To assess potential synergistic interactions between *ethA1* and *ethA2*, three triple knockout strains (ΔΔ*ethA1*Δ*ethA2*, ΔΔ*ethA3*Δ*ethA1*, and ΔΔ*ethA3*Δ*ethA2*) were generated from their corresponding double knockout parental strains (Fig. S8). Simultaneous deletion of *ethA1* and *ethA2* in Δ*nudC* generated strain ΔΔ*ethA1*Δ*ethA2*, which exhibited high-level resistance to ETH, with its MIC restored to the WT level of 256 μg/mL (Table 2). This restoration indicates synergistic epistasis between *ethA1* and *ethA2*. The triple knockout strains ΔΔ*ethA3*Δ*ethA1* and ΔΔ*ethA3*Δ*ethA2* exhibited ETH susceptibility levels identical to those of the ΔΔ*ethA1* and ΔΔ*ethA2* strains, respectively (Table 2). This result aligns with our prior observation that deletion of *ethA3* does not further alter ETH susceptibility beyond the effects of deleting *ethA1* or *ethA2* (Table 2). DST results on solid agar media correlated with MIC values determined in liquid culture (Fig. 3B). Furthermore, deletion of *ethA1*, *ethA2*, or *ethA3* in Δ*nudC* does not affect growth rate (Fig. S3). No significant difference in growth rates was observed among the triple knockout strains ΔΔ*ethA1*Δ*ethA2*, ΔΔ*ethA3*Δ*ethA1*, and ΔΔ*ethA3*Δ*ethA2* (Fig. S3). Collectively, these data indicate that MAB_0985 (EthA1) and MAB_0103 (EthA2) are the most likely functional EthA homologs mediating ETH activation in *M. abscessus*.

### Overexpression of *ethA1*, *ethA2*, or *ethA3* in the Δ*nudC* and WT increased their susceptibility to ETH

**Fig 4:**
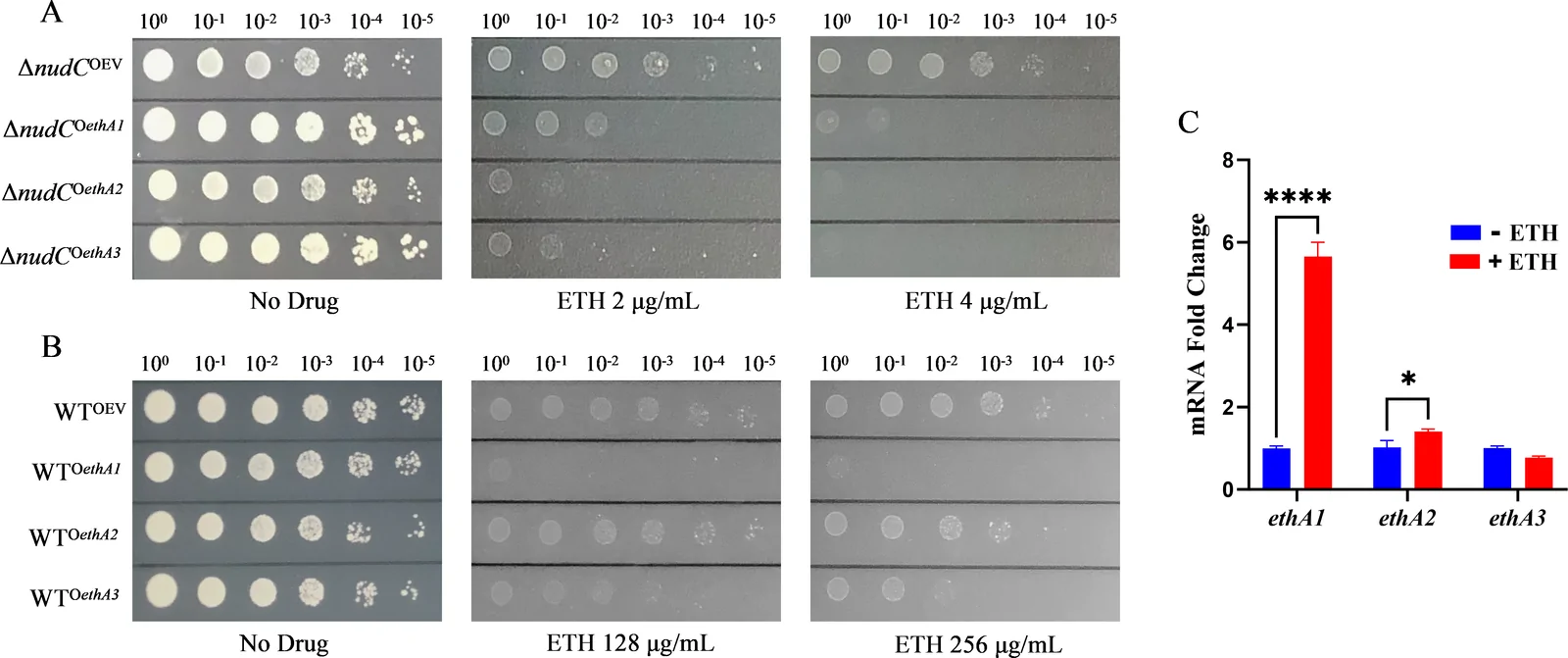
Overexpression of *ethA1* (*MAB_0985*)*, ethA2* (*MAB_0103*), or *ethA3* (*MAB_3967*) in the *nudC* knockout strain or wild-type *M. abscessus* enhances susceptibility to ETH. (**A**) Susceptibility of *nudC* knockout strains overexpressing *ethA1*, *ethA2,* or *ethA3* to ETH. Δ*nudC*^OEV^, Δ*nudC*^O*ethA1*^, Δ*nudC*^O*ethA2*^, and Δ*nudC*^O*ethA3*^, *M. abscessus nudC* knockout strain containing an empty vector or the vector for overexpressing *ethA1*^Mab^, *ethA2*^Mab^, or *ethA3*^Mab^, respectively. (**B**) Susceptibility of wild-type *M. abscessus* overexpressing *ethA1*, *ethA2,* or *ethA3* to ETH. WT^OEV^, WT^O*ethA1*^, WT^O*ethA2*^, and WT^O*ethA3*^, wild-type *M. abscessus* containing an empty vector or the vector for overexpressing *ethA1*^Mab^, *ethA2*^Mab^, or *ethA3*^Mab^, respectively. (**C**) Gene expression levels of *ethA1*, *ethA2*, and *ethA3* in the *nudC* knockout strain in the presence and absence of ETH. *P < 0.05 and ****P < 0.0001. Data are means ± SD of three biological replicates.

To further investigate the contribution of EthA proteins to ETH activation, *ethA1*, *ethA2*, and *ethA3* were individually overexpressed from the multicopy, extrachromosomal plasmid pMV261 in the Δ*nudC* background. Overexpression of each gene increased ETH susceptibility, reducing MIC values to 2 μg/mL for Δ*nudC*^O*ethA1*^ and Δ*nudC*^O*ethA3*^ and 1 μg/mL for Δ*nudC*^O*ethA2*^. These changes corresponded to 8-, 8-, and 16-fold reductions in MIC relative to the parental strain, respectively (Table 2; Table S2). Similarly, on solid agar plates, Δ*nudC*^O*ethA1*^, Δ*nudC*^O*ethA2*^, and Δ*nudC*^O*ethA3*^ were susceptible to ETH at 2 μg/mL, which is consistent with the liquid MIC results (Fig. 4A). The growth rates of these overexpressed strains were not affected (Fig. S3). To further validate their functions, we overexpressed *ethA1*, *ethA2*, or *ethA3* in WT. The MIC of ETH to the WT^O*ethA1*^ was 128 μg/mL, representing a twofold decrease compared to the parental WT (Table 2). In contrast, no change in the MIC of ETH was observed for the WT^O*ethA2*^ and WT^O*ethA3*^. However, on agar plates, the WT^O*ethA1*^ exhibited sensitivity to ETH at 128 μg/mL, while the WT^O*ethA2*^ and WT^O*ethA3*^ displayed slight sensitivity at 256 μg/mL. This indicates that overexpression of *ethA2* and *ethA3* in WT confers detectable increased susceptibility to ETH (Fig. 4B). Quantitative reverse transcription-PCR (qRT-PCR) analysis demonstrated that, following 4-h stimulation with 16 μg/mL ETH, the mRNA level of *ethA1* in Δ*nudC* increased 5.65-fold, whereas *ethA2* expression exhibited a modest 1.40-fold increase (Fig. 4C). Conversely, *ethA3* transcript levels exhibited no significant upregulation (Fig. 4C). Analysis of drug sensitivity profiles from the Δ*nudC*^O*ethA3*^, Δ*ethA3*, ΔΔ*ethA3*, ΔΔ*ethA3*Δ*ethA1,* and ΔΔ*ethA3*Δ*ethA2* strains suggests that MAB_3967 (EthA3) may also function as a potential EthA in *M. abscessus*. Its role in ETH activation may only become detectable under conditions of substantial overexpression.

### Deletion of *ethA1* or *ethA2* reduces the formation of the ETH-NAD adduct in Δ*nudC* treated with ETH

Our prior research demonstrated that *nudC* deletion in WT resulted in the accumulation of an ETH-NAD adduct, which significantly increased susceptibility to ETH. Therefore, we employed high-performance liquid chromatography-mass spectrometry to quantify ETH-NAD adduct levels in the double and triple knockout strains to determine the contribution of EthA1 and EthA2 to ETH activation. HPLC-MS analysis revealed that under 256 μg/mL ETH treatment, the levels of ETH-NAD adduct in both double knockout strains (ΔΔ*ethA1* and ΔΔ*ethA2*) were significantly reduced, with peak area ratios approximately 0.23- and 0.61-fold of that in the Δ*nudC*, respectively. Moreover, the triple knockout strain ΔΔ*ethA1*Δ*ethA2* exhibited an even more pronounced reduction of ETH-NAD adduct, with a peak area ratio of approximately 0.10-fold relative to that from Δ*nudC* (Fig. 1C). In contrast, deletion of *ethA3* in Δ*nudC*, ΔΔ*ethA1*, or ΔΔ*ethA2* did not alter ETH-NAD adduct production. These findings align with the DST results, confirming that EthA1 and EthA2 serve as the principal EthA enzymes in *M. abscessus*.

### EthR inhibits *ethA1* expression but does not influence *ethA2* and *ethA3* expression

**Fig 5:**
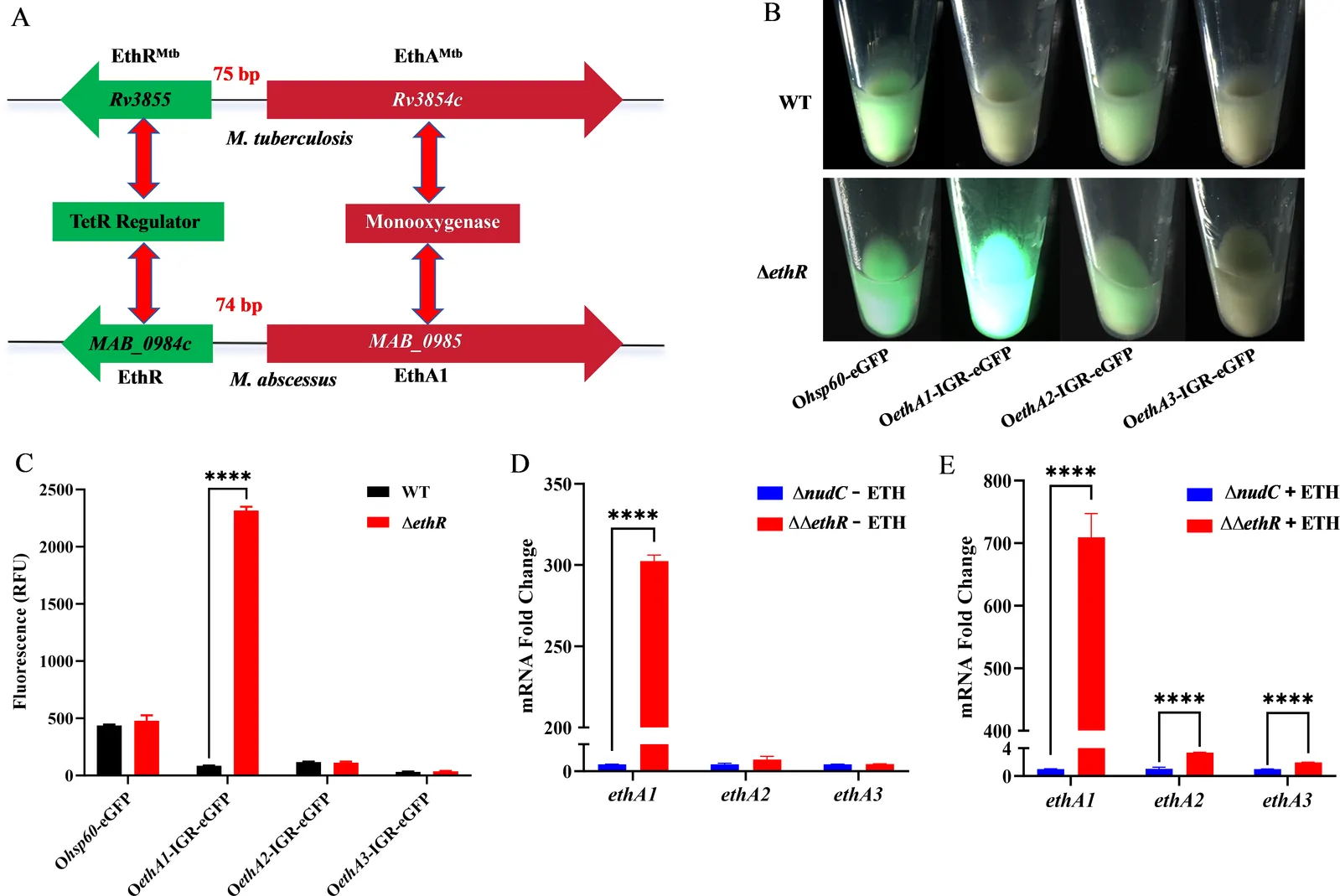
EthR specifically suppresses *ethA1* expression but does not influence *ethA2* and *ethA3* expression. (**A**) Comparison of the genetic organization and predicted protein functions at homologous loci between *M. abscessus* and *M. tuberculosis*. Genes *MAB_0984c* and *MAB_0985* correspond to *ethR*^Mtb^ (*Rv3855*) and *ethA*^Mtb^ (*Rv3854c*), respectively. (**B and C**) Overexpression of the *ethA1*-intergenic region-eGFP in *ethR* knockout strain resulted in a significant increase in relative fluorescence (RFU). WT, wild-type *M. abscessus*; Δ*ethR*, *ethR* knockout strain; O*hsp60*-eGFP, O*ethA1*-IGR-eGFP, O*ethA2*-IGR-eGFP, and O*ethA3*-IGR-eGFP, overexpressing *hsp60*-eGFP, *ethA1* (*MAB_0985*)-IGR-eGFP, *ethA2* (*MAB_0103*)-IGR-eGFP, and *ethA3* (*MAB_3967*)-IGR-eGFP in WT and Δ*ethR*, respectively. (**D and E**) Gene expression levels of *ethA1*, *ethA2*, and *ethA3* in the *ethR* and *nudC* double knockout strain in the absence and presence of ETH. Δ*nudC*, *M. abscessus nudC* knockout strain; ΔΔ*ethR*, *ethR* and *nudC* double knockout strain; ****P < 0.0001. Data are means ± SD of three biological replicates.

The *ethR* (*MAB_0984c*) is located adjacent to the *ethA1* (*MAB_0985*) locus. The genetic organization of the *MAB_0984c-MAB_0985* locus is identical to that of the *M. tuberculosis ethR-ethA* (*Rv3854c-Rv3855*) locus, with both comprising adjacent open reading frames separated by short intergenic regions (IGR, 75 and 74 bp, respectively) (Fig. 5A). Structural homology with the *M. tuberculosis ethA-ethR* regulon indicates that EthR acts as a repressor of *ethA1* expression, likely by modulating the activity of its IGR. Bioinformatic analysis of the IGR between *ethR* and *ethA1* identified consensus −10 and −35 promoter elements, confirming that this IGR functions as the promoter for *ethA1* (Fig. S9A). IGR-enhanced green fluorescent protein (eGFP) reporter strains were engineered to monitor EthR-mediated suppression of *ethA1*. Quantitative analysis of reporter strains in WT demonstrated that fluorescence signals from *ethA1-*, *ethA2-*, and *ethA3*-IGR-eGFP fusion constructs were markedly less intense than those elicited by the *hsp60*-eGFP positive control. However, overexpression of the *ethA1*-IGR-eGFP reporter in the Δ*ethR* mutant increased fluorescence by more than 27-fold relative to WT levels, producing a signal exceeding that of the *hsp60*-eGFP positive control (Fig. 5B and C). In contrast, no significant change in eGFP fluorescence intensity was observed for the *ethA2*-IGR-eGFP or *ethA3*-IGR-eGFP reporters in the Δ*ethR* mutant compared to the WT (Fig. 5B and C). Following *ethR* deletion in the Δ*nudC* strain, *ethA1* transcript levels increased by 302.42-fold (quantified by qRT-PCR), whereas *ethA2* and *ethA3* expression remained unchanged (Fig. 5D). Following treatment with 0.125 μg/mL ETH, *ethA1* mRNA level in the ΔΔ*ethR* increased by 709.32-fold, significantly exceeding the minimal changes observed for *ethA2* (3.37-fold) and *ethA3* (1.96-fold) (Fig. 5E). These findings demonstrate that EthR specifically suppresses *ethA1* expression, while leaving *ethA2* and *ethA3* expression unaffected. Accordingly, *ethR* deletion significantly upregulated *ethA1* expression.

### Molecular docking predicts stable binding of BDM31343 to EthR

**Fig 6:**
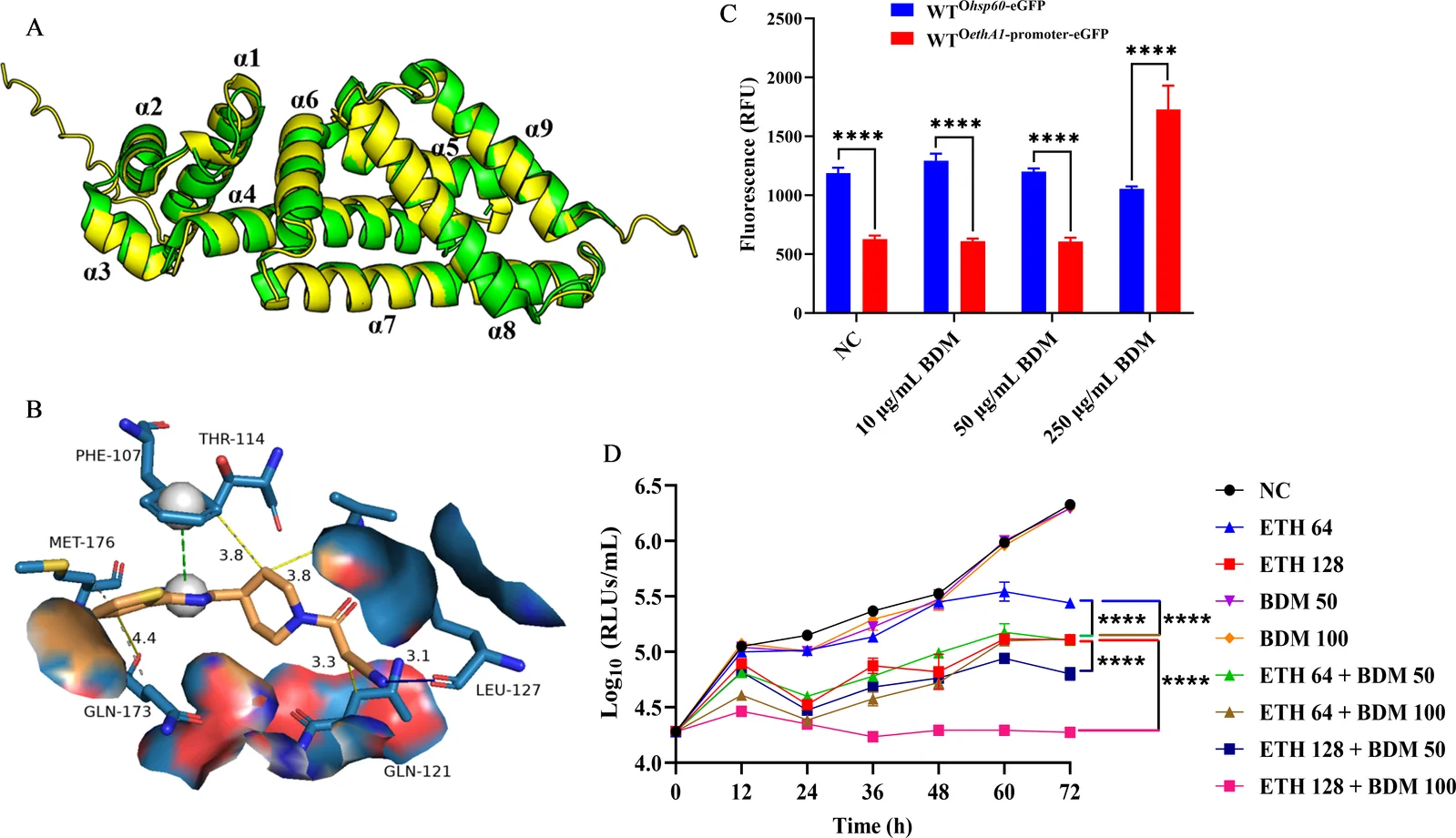
EthR^Mtb^ inhibitor BDM31343 can boost the efficacy of ETH against *M. abscessus* via suppression of EthR. (**A**) The structure of EthR in *M. abscessus* is highly homologous to that of EthR^Mtb^ (PDB code: 3TP0). Green, EthR^Mtb^ structure; yellow, EthR^Mab^ structure predicted by AlphaFold3. (**B**) Interaction patterns between BDM31343 and the amino acid residues of EthR. Blue line, hydrogen bond; green line, π-π stacking interaction; and yellow line, hydrophobic interaction. (**C**) High doses of BDM31343 (BDM) resulted in a significant increase in the relative fluorescence (RFU) in wild-type *M. abscessus* overexpressing *ethA1*-promoter-eGFP. WT^O*hsp60*-eGFP^ and WT^O*ethA1*-promoter-eGFP^, wild-type *M. abscessus* overexpressing *hsp60*-eGFP and *ethA1*-promoter-eGFP, respectively. NC, negative control. ****P < 0.0001. (**D**) Time-kill curves of BDM31343 combined with ETH against autoluminescent *M. abscessus* (AlMab). AlMab cultures (approximately 1 × 10^4^ relative light units [RLUs]/mL) were treated with BDM31343 (50 or 100 μg/mL) in the presence of ETH (64 or 128 μg/mL). Bacterial survival was monitored by measuring RLUs every 12 h for up to 72 h. Numbers following the ETH and BDM indicate the working concentrations (µg/mL). ****P < 0.0001.

The three-dimensional structure of EthR was computationally predicted using AlphaFold3 and validated via a per-residue confidence score (pLDDT) of 88.4, indicating high model reliability (Fig. 6A). Moreover, the predicted structure of EthR exhibits high homology to that of EthR^Mtb^ (PDB code: 3TP0), with both proteins containing nine alpha-helices (Fig. 6A). The predicted dimeric EthR structure of *M. abscessus* aligns closely with that of *M. tuberculosis* (root-mean-square deviation = 1.43 Å), supporting the reliability of the homology model at the quaternary level and suggesting that the two proteins may share similar biological functions (Fig. S10). BDM31343 is an inhibitor of EthR^Mtb^, which increases the antibacterial efficacy of ETH against *M. tuberculosis* by 10-fold (15). To elucidate the potential binding of BDM31343 to EthR homodimer, molecular docking was performed to predict the binding affinity and specific interaction modes. The molecular docking result shows that the inhibitor BDM31343 fits well into the binding pocket of the EthR and is stabilized by multiple non-covalent interactions. The predicted binding free energy for the BDM31343-EthR^Mab^ complex is −9.34 kcal/mol, compared with −10.32 kcal/mol for BDM31343-EthR^Mtb^, suggesting a weaker affinity of BDM31343 for EthR^Mab^. The binding is anchored by a specific hydrogen bond with LEU-127 (Fig. 6B). This conformation is further stabilized by strong π-π stacking interactions with PHE-107 and hydrophobic contacts involving residues such as THR-114, GLN-121, GLN-173, and MET-176 (Fig. 6B). This synergistic interplay of interactions underlies the potent and specific binding affinity of BDM31343, enabling its effective inhibition of the EthR protein.

### BDM31343 potentiates ETH activity against *M. abscessus* via inhibition of EthR

To further evaluate BDM31343’s inhibitory effect on EthR, the compound was administered to cultures of the IGR-eGFP reporter strain. When the concentration of BDM31343 reached 250 μg/mL, a marked increase in the *ethA1* promoter activity was observed, resulting in the eGFP fluorescence intensity surpassing even that of the *hsp60*-eGFP control (Fig. 6C). To further validate the efficacy of BDM31343, we employed an autoluminescent *M. abscessus* (AlMab) (18) strain to assess whether this compound potentiates the antibacterial activity of ETH against *M. abscessus*. Both concentrations of BDM31343 (50 and 100 μg/mL) produced a 0.33 log_10_ relative light unit (RLU) decrease in AlMab under 64 μg/mL ETH, with growth inhibition equivalent to 128 μg/mL ETH treatment (Fig. 6D). At 128 μg/mL ETH, the reduction in AlMab RLUs was greater at 100 μg/mL BDM31343 (0.83 log_10_) than at 50 μg/mL (0.31 log_10_), while complete growth inhibition occurred only at the higher concentration (Fig. 6D). These findings demonstrate that high-dose BDM31343 inhibits EthR and enhances *M. abscessus* susceptibility to ETH, albeit with reduced efficacy compared to its activity against *M. tuberculosis*. Therefore, developing small-molecule inhibitors targeting *M. abscessus* EthR represents a promising strategy to enhance the antibacterial efficacy of ETH.

## DISCUSSION

*M. abscessus* is a predominant rapidly growing NTM causing both pulmonary and extrapulmonary infections. The intrinsic resistance of *M. abscessus* to antibiotics, arising from both its highly impermeable cell envelope and diverse resistance mechanisms, severely limits treatment options and contributes to poor clinical outcomes (19). Therefore, elucidating the molecular mechanisms of drug resistance in *M. abscessus* is critically important for clinical management. Given this bacterium’s unique resistance profile and poor treatment outcomes, the identification of novel drug targets and resistance-modulating strategies remains an urgent priority.

ETH is one of the most effective second-line drugs for the treatment of multidrug-resistant tuberculosis. However, its clinical utility is limited by dose-dependent adverse effects, including gastrointestinal toxicity, which often compromise treatment adherence (15). Strategies that enable ETH dose reduction while preserving antibacterial efficacy are therefore highly desirable. In *M. tuberculosis*, ETH is activated by the Baeyer-Villiger monooxygenase (BVMO) EthA (11, 20, 21), resulting in a stable ETH-NAD adduct, which subsequently inhibits the InhA, thereby disrupting mycolic acid biosynthesis (22, 23). Since the expression of EthA is regulated by the TetR-family transcriptional repressor EthR (24), small-molecule inhibitors of EthR have been successfully developed for *M. tuberculosis* to stimulate EthA expression (15, 25–28), thereby potentiating ETH activation and enhancing its antibacterial activity (14, 15). Although EthR and EthA are well characterized in *M. tuberculosis*, their homologous counterparts and associated functions in *M. abscessus* are yet to be elucidated. In this study, we identify EthR and its cognate target EthA1 in *M. abscessus* for the first time. Furthermore, we demonstrated that disrupting this regulatory mechanism increases susceptibility to ETH.

It is well established that homologous proteins with amino acid sequence identity exceeding 40% are highly likely to share functional similarity (29). BLASTp analysis using the EthA^Mtb^ sequence identified three putative homologs in *M. abscessus*: MAB_0985, MAB_0103, and MAB_3967, which we designated as EthA1, EthA2, and EthA3, respectively. Each shares over 40% amino acid sequence identity with EthA^Mtb^. Functional characterization demonstrated that EthA1 and EthA2 are the principal enzymes responsible for ETH activation in *M. abscessus*. Disruption of either gene in Δ*nudC* significantly reduced ETH-NAD adduct formation and consequently enhanced bacterial resistance to ETH. Remarkably, the combined deletion of *ethA1* and *ethA2* in the Δ*nudC* abolished ETH activation in the resulting triple mutant. This reduction in ETH-NAD adduct formation conferred high-level ETH resistance.

Moreover, overexpression of either *ethA1* or *ethA2* in WT and Δ*nudC* enhanced susceptibility to ETH. Overexpression of *ethA1* or *ethA2* conferred greater ETH sensitivity in Δ*nudC* than in WT, probably due to hydrolysis of the ETH-NAD adduct by NudC in WT. Following treatment with ETH, the Δ*nudC* mutant exhibited a significant upregulation of *ethA1* transcription and a modest increase in *ethA2* expression. However, as a prodrug, ETH does not directly activate *ethA* transcription (21, 24). We hypothesize that the accumulation of ETH-NAD adduct in Δ*nudC* may indirectly relieve EthR-mediated repression, possibly through feedback modulation or by perturbing the intracellular metabolic environment, thereby affecting the DNA-binding activity of EthR. The precise mechanism by which ETH-NAD adduct accumulation leads to *ethA1* or *ethA2* upregulation remains to be elucidated.

**Fig 7:**
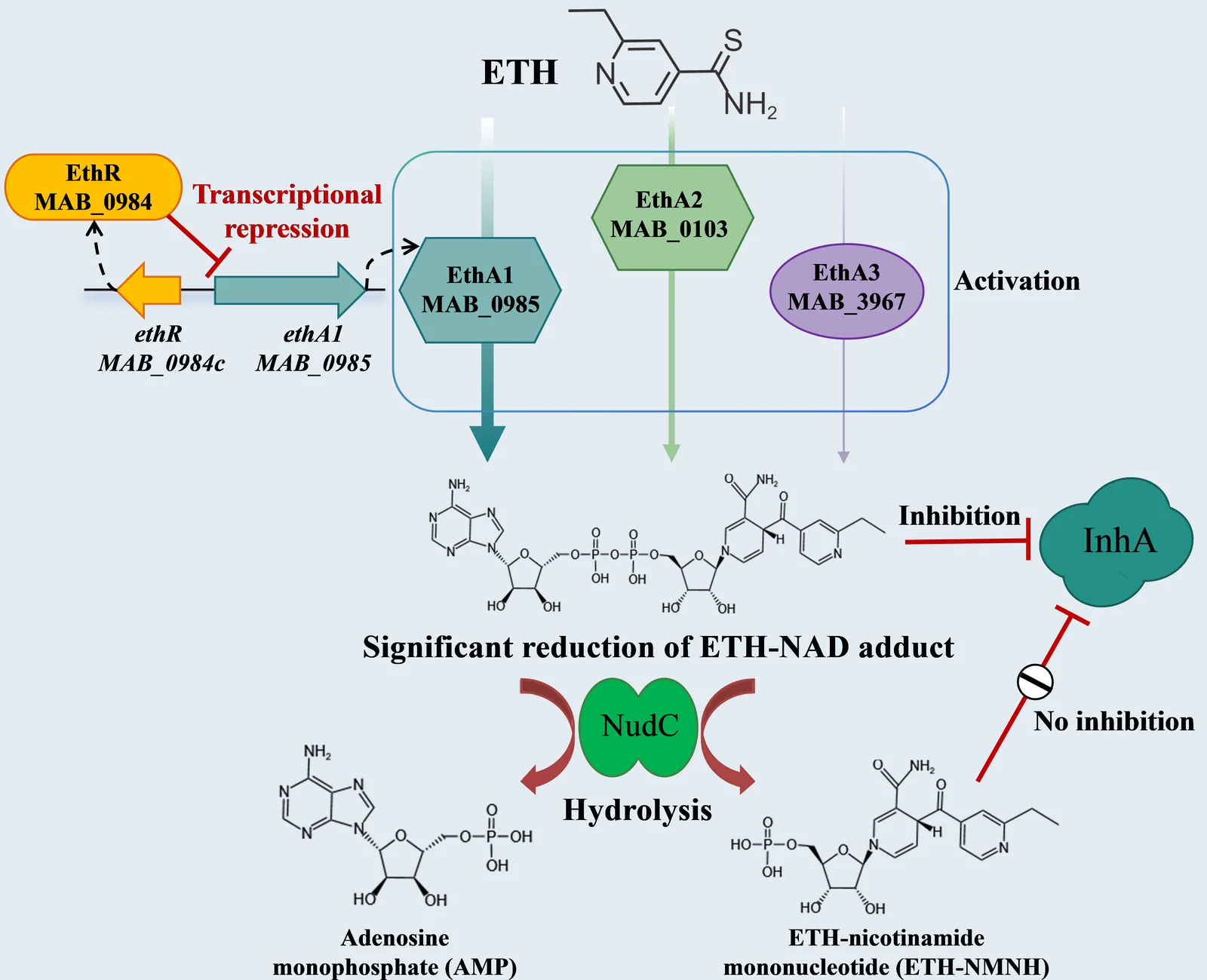
Schematic representation of the resistance mechanism of *M. abscessus* to ETH. ETH is activated by EthA1 (MAB_0985), EthA2 (MAB_0103), and EthA3 (MAB_3967) to form the ETH-NAD adduct in *M. abscessus*. Among them, EthA1 exhibits the strongest activating potency, followed by EthA2, while EthA3 is the weakest. EthR specifically suppresses *ethA1* expression, resulting in a significant reduction of ETH-NAD adduct in *M. abscessus*. The ETH-NAD adduct can be hydrolyzed by NudC into adenosine monophosphate (AMP) and ETH-nicotinamide mononucleotide (ETH-NMNH). This reaction eliminates the adduct’s inhibition of InhA, consequently conferring resistance to ETH in *M. abscessus*. The thickness of the lines connected to ETH represents the ETH-activating capability.

Conversely, deletion of *ethA3* did not significantly affect susceptibility to ETH in either the WT or Δ*nudC* strain. No significant change in *ethA3* expression was observed in the Δ*nudC* following ETH treatment. Collectively, these data imply that EthA3 does not appear to be a major EthA in *M. abscessus*. However, overexpression of *ethA3* in the Δ*nudC* using the multicopy plasmid pMV261 conferred high susceptibility to ETH. Therefore, EthA3 should be regarded as a functional EthA in *M. abscessus*, yet its physiological role appears minimal under native conditions. Functional significance emerges only upon overexpression, likely due to its inherently low catalytic efficiency. Therefore, EthA1 exhibits the strongest ETH-activating capability, followed by EthA2, while EthA3 is the weakest (Fig. 7). Moreover, the conserved BVMO motif [FXGXXXHXXXW(P/D)] (30), characteristic of EthA in *M. tuberculosis*, was identified in EthA1, EthA2, and EthA3 of *M. abscessus* (Fig. S4). Under physiological conditions, BMVOs are primarily involved in oxidative degradation of endogenous substrates such as ketones and steroids (31). The differential activation efficiency of ETH by EthA1, EthA2, and EthA3 likely arises from their distinct substrate specificities, as each BVMO typically exhibits selectivity toward specific compound classes (13), and ETH is not a natural substrate for BMVOs. Like *ethA1*, the *ethA2* and *ethA3* genes are likely regulated by their cognate EthR protein. However, the genes adjacent to *ethA2* and *ethA3* do not belong to either the TetR family of repressors or the AraC/XylS family of transcriptional factors, indicating that their specific transcriptional regulators remain to be identified.

Our previous study revealed a mechanism of ETH resistance in *M. abscessus* mediated by NudC, encoded by *MAB_3513c* (16). NudC functions as a pyrophosphohydrolase that hydrolyzes the ETH-NAD adduct, thereby counteracting ETH toxicity. The proline residue at position 226 was shown to be critical for NudC dimerization and hydrolase activity, which underlies the intrinsic ETH resistance. However, the *nudC* knockout reduced the MIC of ETH against *M. abscessus* from 256 to 16 μg/mL, which was still higher than the MIC of ETH for *M. tuberculosis*, suggesting the existence of additional resistance mechanisms in *M. abscessus*. In *M. tuberculosis*, CinA has been shown to confer cross-tolerance to INH, ETH, delamanid, and pretomanid by hydrolyzing drug-NAD adducts, functioning in a manner similar to NudC (32). Whether a CinA homolog in *M. abscessus* contributes to ETH resistance, in addition to NudC, has not been reported. However, knockout of *MAB_2443*, the putative *cinA* homolog, in either the WT or Δ*nudC* strain did not affect *M. abscessus* susceptibility to ETH or INH (Table 2). These results indicate that CinA^Mab^ does not fulfill the same functional role as CinA^Mtb^, and the underlying mechanism warrants further investigation.

In this study, we identified MAB_0984 as an additional key determinant of ETH resistance in *M. abscessus*, which functions as EthR by inhibiting the expression of *ethA1* to reduce ETH activation. Bioinformatic analysis revealed that *ethA1* is adjacent to the TetR-family transcriptional regulator gene *MAB_0984c*, mirroring the genomic organization of the *ethR-ethA* locus (*Rv3854c-Rv3855*) in *M. tuberculosis*. MAB_0984 exhibits significant sequence identity with *M. tuberculosis* EthR, strongly supporting its classification as a functional homolog of this transcriptional regulator. The increased susceptibility to ETH observed upon *ethR* knockout was substantially greater than that resulting from *nudC* deletion, indicating EthR’s more critical role in conferring intrinsic resistance. Concurrent disruption of *nudC* and *ethR* produced a synergistic effect, reducing the MIC substantially below that observed with either single knockout. Metabolite analysis directly links the observed phenotypic differences to underlying mechanistic changes. While *nudC* disruption impairs adduct clearance, leading to moderate accumulation, the significantly greater buildup in the ΔΔ*ethR* mutant reflects profound dysregulation of the ETH activation pathway. These findings demonstrate that intrinsic ETH resistance in *M. abscessus* is primarily governed by the EthR-regulated activation pathway rather than the NudC-mediated clearance pathway. Disrupting EthR substantially enhances susceptibility to ETH, establishing it as the primary drug target. However, complete reversal of resistance necessitates dual targeting of both pathways, as inhibition of either mechanism alone is insufficient to overcome this robust intrinsic barrier. Mechanistic studies using qRT-PCR and an IGR-eGFP reporter system confirmed that EthR inhibits *ethA1* expression by regulating the activity of the promoter. The nucleotide sequence of the *ethA1*^Mab^ promoter shares 41.98% identity with that of the *ethA*^Mtb^ promoter (Fig. S9B). Notably, the key nucleotides involved in EthR binding are highly conserved between the two species (24), implying a highly similar regulatory mechanism for *ethA1* by EthR in *M. abscessus*. Collectively, these results demonstrate that MAB_0984 functions as the EthR regulator in *M. abscessus*, which inhibits EthA1 production and thereby mediates intrinsic resistance to ETH (Fig. 7). In addition, the 8-fold reduction in the ETH MIC observed upon *ethA1* overexpression was substantially smaller than the 64-fold reduction resulting from *ethR* deletion. The expression level of *ethA1* was increased by 86.16-fold in the overexpression strain (Fig. S11), which was markedly lower than the 302.42-fold upregulation of *ethA1* observed upon *ethR* deletion. This discrepancy is not unexpected, as *ethR* deletion relieves transcriptional repression of the endogenous *ethA1* locus, enabling robust gene expression within its native regulatory context. In contrast, plasmid-based overexpression may be limited by downstream cellular constraints, including translational capacity, protein folding efficiency, and the availability of essential cofactors required for EthA1 activity.

Small-molecule compounds targeting EthR^Mtb^ have been found to significantly boost the efficacy of ETH both *in vitro* and *in vivo*. Co-administration of the EthR-targeting compounds BDM31343 or BDM41906 with ETH dramatically lowers the required ETH dose without compromising efficacy, achieving mycobacterial load comparable to standard high-dose treatment (15, 28). However, these compounds failed to enhance the activity of ETH against *ethA*-mutant strains. Subsequently, EthA2 and EthR2 were identified in *M. tuberculosis*, prompting the development of SMARt420, a specific inhibitor targeting EthR2 (12). SMARt420 not only completely reversed acquired ETH resistance and eradicated ETH-resistant bacterial loads in mice but also enhanced the baseline susceptibility of *M. tuberculosis* to ETH. The recently identified compound SMARt751 targets the *virS* transcriptional regulator in *M. tuberculosis* to modulate the *mymA* operon, which encodes a monooxygenase capable of activating ETH (14). SMARt751 significantly boosted the therapeutic efficacy of ETH in both *in vitro* assays and murine models of acute/chronic tuberculosis. Moreover, it restored the full therapeutic efficacy of ETH in mice infected with ETH-resistant strains. SMARt751 uniquely circumvents the predominant resistance mechanisms found in clinical *M. tuberculosis* isolates, such as alterations in the *ethA, ethR* promoter, and *inhA* promoter, thereby overcoming typical forms of ETH resistance. However, no homologs of EthR2 or VirS could be identified in *M. abscessus*. The EthR^Mtb^ inhibitor BDM31343 exhibits cross-species activity by inhibiting EthR^Mab^, as shown by increased eGFP signal in *M. abscessus* containing an *ethA1-*promoter-eGFP construct. BDM31343 enhances the efficacy of ETH against *M. abscessus*, albeit at elevated concentrations. It is worth noting that the concentration of BDM31343 required to potentiate ETH activity in *M. abscessus* (100 μg/mL) is substantially higher than that typically effective in *M. tuberculosis*. This disparity likely arises from two main factors. First, the *M. abscessus* cell envelope is notoriously impermeable due to its highly hydrophobic mycolic acid outer membrane and surface-exposed glycopeptidolipids, which together impede the intracellular accumulation of small molecules (33). Second, although EthR^Mab^ is a functional homolog of EthR^Mtb^ (50.23% identity), sequence divergence within the ligand-binding pocket may reduce the binding affinity of BDM31343 for EthR^Mab^. This is supported by our molecular docking analysis, which predicts altered interactions compared to the EthR^Mtb^-BDM31343 co-crystal structure. Thus, while BDM31343 validates EthR as a druggable target in *M. abscessus*, it is not suitable for clinical use. Future efforts should focus on developing *M. abscessus*-specific EthR inhibitors with improved potency, optimized cell wall permeability, and robust target engagement, along with a favorable safety profile. Moreover, once such improved inhibitors are developed, their therapeutic potential should be systematically evaluated against a diverse panel of clinical isolates and further validated in infected macrophage models and animal infection models. Such an approach represents a potential strategy to effectively potentiate ETH-based therapy against *M. abscessus*.

In conclusion, this study identifies MAB_0984 as the functional EthR transcriptional repressor, which specifically suppresses *ethA1* expression, resulting in a significant reduction of the ETH-NAD adduct in *M. abscessus*. The intrinsic resistance of *M. abscessus* to ETH is primarily governed by the EthR-regulated activation pathway rather than the NudC-mediated clearance pathway. We further demonstrate that EthA1 (MAB_0985) and EthA2 (MAB_0103) function as the principal ETH-activating monooxygenases, whereas EthA3 (MAB_3967) contributes minimally under physiological conditions. The development of EthR inhibitors represents a promising avenue to sensitize mycobacteria to ETH. This work elucidates the mechanism of ETH resistance in *M. abscessus*, establishing a conceptual and mechanistic framework for developing more effective ETH-based therapies.

## MATERIALS AND METHODS

### Bacterial strains and culture conditions

*Escherichia coli Trans* T1 (Beijing TransGen Biotech) was used for plasmid propagation and cultured at 37°C in liquid or solid LB medium. The strain *M. abscessus* GZ002 (NCBI GenBank accession number CP034181), isolated and characterized in our laboratory, was used as the parental strain throughout this study. It was cultured at 37°C in 7H9 broth (containing 2% glycerol, 10% OADC, and 0.05% Tween 80) or on 7H10 agar (supplemented with 2% glycerol and 10% OADC). When necessary, kanamycin (Kan, 50 μg/mL) or zeocin (Zeo, 30 μg/mL) was added to the medium for plasmid selection and maintenance. Anhydrotetracycline (ATc) was used as an inducer at a concentration of 100 ng/mL. The autoluminescent *M. abscessus* strain, engineered in our laboratory, was used for drug susceptibility testing based on luminescence readouts.

### Identification, knockout, and complementation of putative *ethR* and *ethA* homologs

Putative *ethR* and *ethA* homologs in *M. abscessus* were identified by BLASTp analysis using characterized EthR and EthA proteins from *M. tuberculosis* as query sequences. Genes exhibiting >40% amino acid sequence identity were selected for functional analysis.

Gene knockouts were generated using a CRISPR-NHEJ system. Briefly, the temperature-sensitive plasmid pCR-Zeo (generously provided by Prof. Yicheng Sun’s laboratory) was utilized for crRNA expression to generate knockout mutants. A 5′-TTC-3′ protospacer adjacent motif was selected for target recognition. Three independent crRNAs targeting the 5′ region of the putative *ethR* and *ethA* genes were designed (Table S3). Gene-specific oligonucleotides containing the crRNA sequences were synthesized, annealed, and ligated into the linearized pCR-Zeo vector. The resulting recombinant plasmids were introduced by electroporation into *M. abscessus* strains harboring the pNHEJ-cpf1 plasmid.

Transformants were selected on Middlebrook 7H11 agar supplemented with Kan (100 μg/mL), Zeo (30 μg/mL), and ATc (200 ng/mL), followed by incubation at 30°C for 72 h. Single colonies were screened by PCR, followed by agarose gel electrophoresis and Sanger sequencing (performed by Sangon Biotech, Shanghai) to confirm the construction of the desired knockout strains. For genetic complementation, the corresponding wild-type genes were amplified and cloned into the multicopy, extrachromosomal plasmid pMV261. Recombinant plasmids were introduced into the respective deletion mutants by electroporation, and successful complemented strains were confirmed by PCR and DNA sequencing.

### DST

The MICs were determined by the broth microdilution method. WT and the putative *ethA* and *ethR* knockout and complemented strains were first grown in 7H9 medium to the mid-log phase (OD_600_ = 0.6–0.8). The cultures were then diluted to an OD_600_ of 0.1–0.2 in Tween-80-free 7H9 medium and subsequently diluted 1,000-fold to prepare the final inoculum for testing. The drugs were subjected to twofold serial dilution. The assay was performed with four technical replicates for each concentration in three independent biological replicates. MICs were defined as the lowest drug concentration that completely inhibited visible bacterial growth. We performed DST on solid medium by spotting 10-fold serial dilutions (up to 10^−5^) of mid-log-phase bacterial cultures onto 7H10 agar plates containing specified drug concentrations. Antibiotic-free plates were used as negative controls, and all assays were repeated three times.

### Determination of growth curves

Bacterial growth was monitored by measuring the absorbance at 600 nm. Mid-exponential phase cultures of *M. abscessus* were diluted to an OD_600_ of 0.05 and incubated at 37°C with shaking at 200 rpm. The OD_600_ was measured at 6-h intervals for a total duration of 72 h using a spectrophotometer. Experiments were performed in triplicate, and the growth curves were plotted using GraphPad Prism 10.1.2.

### Extraction of ETH metabolites

ETH metabolites were extracted as previously described (16), with minor modifications. Briefly, bacterial strains were cultured to an OD_600_ of 0.7 and then exposed to 256 μg/mL ETH for 3 h at 37°C in a shaking incubator. Bacteria were harvested and washed twice with deionized water by centrifugation at 13,000 × *g* for 10 min at 4°C. The obtained bacterial precipitate was resuspended in 6 mL of chloroform-methanol (2:1), incubated at room temperature with shaking at 200 rpm for 3 h, followed by solvent evaporation using a CentriVap concentrator (Labconco). The dried bacterial pellet was resuspended in 100 mM ammonium carbonate (pH 7.0) and subjected to vigorous mixing with 0.1 and 0.5 mm zirconia beads for physical disruption. The lysate was centrifuged at 18,000 × *g* for 20 min at 4°C, and the resulting supernatant was collected and passed through a 0.22 μM filter (Millipore) to remove cellular debris and large particles. The final filtrate was acidified with formic acid to a final concentration of 0.1% (vol/vol), followed by centrifugation at 18,000 × *g* for 20 min at 4°C. Subsequently, a 2 μL aliquot of each resulting product was analyzed by HPLC-MS using an Ultimate 3000/Q-Exactive HF-X system (Thermo Fisher Scientific).

### Detection of ETH-NAD adducts

HPLC-MS analysis was employed to detect the ETH-NAD adduct. Chromatographic separation was performed by injecting 2 μL of sample onto an ACQUITY UPLC BEH C18 column (100 × 2.1 mm, 1.7 μm; Waters). Chromatographic separation was performed at a flow rate of 0.3 mL/min using a linear gradient of 0.1% formic acid in water (mobile phase A) and acetonitrile (mobile phase B): 1%–90% B over 4 min, followed by 90% B for 1 min and re-equilibration at 1% B for 2 min. MS detection was conducted on an Ultimate 3000/Q Exactive HF-X system (Thermo Fisher Scientific) in positive ion mode over an *m/z* range of 100–1,000 with working parameters as follows: sheath gas flow rate, 40; auxiliary gas flow rate, 10; spray voltage, 3.5 kV; capillary temperature, 300°C; S-lens RF level, 55; and auxiliary gas heater temperature, 350°C.

The identity of the ETH-NAD adduct was confirmed based on three criteria: (i) an observed protonated molecular ion matching the predicted *m/z* value of 799.1848 ([M + H]^+^) with a mass error of <10 ppm; (ii) detection exclusively in ETH-treated samples and absence in untreated controls; and (iii) the presence of diagnostic fragment ions corresponding to adenosine diphosphate (*m/z*, 428.036) and adenine (*m/z*, 136.062), consistent with the predicted structure of the ETH-NAD adduct (16, 32).

### Construction of eGFP reporter strains and fluorescence analysis

To construct the reporter plasmids, the IGR of *ethA1*, *ethA2*, and *ethA3* were amplified and cloned upstream of the *egfp* gene in the integrative plasmid pRH2502. These plasmids were then electroporated into WT and Δ*ethR* strains. The transformation mixture was plated on 7H10 agar plates containing Kan (100 μg/mL) and incubated at 37°C for 72 h. Single colonies were then selected for PCR amplification and subsequent DNA sequencing to confirm the correct constructs, resulting in the corresponding eGFP reporter strains. For qualitative fluorescence analysis, cells from a 10 mL culture of the eGFP reporter strain (OD_600_ = 0.8) were harvested and visualized using a fluorescence microscope (Leica M205 FA). For quantitative analysis, 150 μL aliquots of exponentially growing cultures of the eGFP reporter strain were dispensed in triplicate into white 96-well plates and incubated at 37°C for 72 h. eGFP fluorescence (excitation, 488 nm; emission, 520 nm) was measured using a FlexStation 3 microplate reader (Molecular Devices).

### RNA extraction and qRT-PCR

Δ*nudC* and ΔΔ*ethR* strains were grown to mid-log phase and treated with ETH at their respective MICs (16 and 0.125 μg/mL) for 4 h, with equal volumes of DMSO serving as the negative controls for each treatment. Total RNA was extracted using the HiPure Bacterial RNA Kit (Magen) and quantified via Nanodrop UV spectrophotometry (Thermo Fisher Scientific). RT-qPCR was conducted using the HiScript III All-in-one RT SuperMix Perfect for qPCR (Vazyme) and Taq Pro Universal SYBR qPCR Master Mix (Vazyme) on a CFX96 Touch Real-Time PCR Detection System (Bio-Rad). The primer sequences are listed in Table S3. Quantitative PCR data were analyzed using the 2^−ΔΔ*C*t^ method, with relative mRNA expression of target genes normalized to the reference gene *sigA* (34).

### Molecular docking analysis

AlphaFold3 (https://alphafoldserver.com/) was used to predict the structure of EthR. The chemical structure of BDM31343 was obtained from the NCBI PubChem database (https://pubchem.ncbi.nlm.nih.gov/). Structural alignment of *M. abscessus* EthR with its homologous protein EthR^Mtb^ (PDB code: 3TP0) was performed using PyMOL (http://www.pymol.org). AutoDock Tools version 1.5.6 was employed to predict the binding affinity and interaction modes between BDM31343 and the predicted structure of EthR.

### Evaluation of BDM31343-mediated potentiation of ETH activity using the AlMab

The AlMab was constructed using the *dif*/Xer recombination system previously established in our laboratory (35). Briefly, the integrating plasmid pOPAI1B, carrying the complete *luxCDABE* operon from *Photorhabdus luminescens* driven by the mycobacterial *hsp60* promoter, an *attP* site, and the *dif*-Xer recombination module, was electroporated into *M. abscessus*. After site-specific integration of the *lux* cassette into the chromosome at the *attB* site, the selection marker was excised via Xer recombinase-mediated recombination, generating an unmarked, stable autoluminescent strain. The resulting AlMab strain constitutively emits bioluminescence without exogenous substrate addition, enabling real-time monitoring of bacterial metabolic activity by measuring the RLUs. The characterization and validation of this strain have been described in detail by our lab (18). The AlMab culture was grown to a density of 1 × 10^6^ RLUs and then adjusted to a working concentration of 3,000–5,000 RLUs. In 1.5 mL microcentrifuge tubes, 5 μL of BDM31343 and/or ETH solutions (dissolved in DMSO) was mixed with 195 μL of the diluted AlMab suspension, resulting in a final volume of 200 μL. The mixtures were gently vortexed, mixed thoroughly, and incubated at 37°C. The final concentrations of BDM31343 were 50 or 100 μg/mL, whereas ETH was used at 64 or 128 μg/mL, either alone or in combination. For the negative control, 5 μL of DMSO was added to 195 μL of AlMab. RLU values were monitored every 12 h using a GloMax 20/20 luminometer throughout the 72-h incubation period.

### Statistical analysis

Data were analyzed using GraphPad Prism version 10.1.2 (GraphPad Software, San Diego, CA, USA). Results are presented as means ± standard deviations from at least three independent biological replicates unless otherwise indicated. Statistical significance was assessed using an unpaired Student’s *t*-test or one-way ANOVA, with statistical significance set at *P* < 0.05.

## Data Availability

The data sets supporting the conclusions of this study are included within the article and its supplemental material. All relevant data sets are fully accessible within this publication.

## SUPPLEMENTAL MATERIAL

Supplemental figures and tables spectrum.00638-26-s0001.pdf Fig. S1 to S11 and Tables S1 to S3.

ASM does not own the copyrights to Supplemental Material that may be linked to, or accessed through, an article. The authors have granted ASM a non-exclusive, world-wide license to publish the Supplemental Material files. Please contact the corresponding author directly for reuse.
