# Supplementary material for: Identification and characterization of ethR and ethA genes impacting the sensitivity of Mycobacterium abscessus to ethionamide
Source: Microbiol Spectr. 2026 Aug 28;14(10):e00638-26. doi: 10.1128/spectrum.00638-26 (PMC13689078; doi:10.1128/spectrum.00638-26)
Supplement: Supplementary file 1 [file spectrum.00638-26-s0001.pdf]

Identification and characterization of *ethR* and *ethA* genes impacting the sensitivity of *Mycobacterium abscessus* to ethionamide

Xiaofan Zhang[1,2,3,4], Lijie Li[1,5], Ziwen Lu[1,2,4], Chaohui Duan[3], H.M. Adnan Hameed[1,2,4] Aweke Mulu Belachew[1,2,4] Cuiting Fang[1,4,6,7,8], Xirong Tian[1,4,6,8], Xinyue Wang[7,8], Hongyi Chen[9], Liqiang Feng[2,4], Matthew B. McNeil[10,11], Dongxin Liu[12], Shuai Wang[4*], Tianyu Zhang[1,2,4,6,7,8*]

[1] State Key Laboratory of Respiratory Disease, Guangzhou Institutes of Biomedicine and Health, Chinese Academy of Sciences, Guangzhou 510530, China

[2] University of Chinese Academy of Sciences, Beijing 100049, China

[3] Department of Clinical Laboratory, Sun Yat-sen Memorial Hospital of Sun Yat-sen University, Guangzhou 510120, China

[4] Guangdong-Hong Kong-Macao Joint Laboratory of Respiratory Infectious Diseases, Guangzhou Institutes of Biomedicine and Health, Chinese Academy of Sciences, Guangzhou 510530, China

[5] School of Life Sciences, University of Science and Technology of China, Hefei 230027, China

[6] Guangzhou Medical University-Guangzhou Institutes of Biomedicine and Health Joint School of Life Sciences, Guangzhou Medical University, Guangzhou 511436, China

[7] State Key Laboratory of Respiratory Disease, Guangzhou Chest Hospital, Guangzhou 510095, China

[8] Guangzhou National Laboratory, Guangzhou 510005, China

[9] West China School of Public Health, Sichuan University, Chengdu 610041, China

[10] Department of Microbiology and Immunology, School of Biomedical Sciences, University of Otago, Dunedin 9054, New Zealand

[11] Maurice Wilkins Centre for Molecular Biodiscovery, University of Auckland, Auckland 1042, New Zealand

[12] Chinese Center for Disease Control and Prevention, National Pathogen Resource Center, Beijing 102206, China

Correspondence: Shuai Wang, wshuai1@163.com; Tianyu Zhang,

zhang_tianyu@gibh.ac.

Table S1 The susceptibility of *M. abscessus ethA1* or *ethA2* knockout strains in a *nudC* deletion background, as well as their complemented strains, to ETH and INH.

M. abscessus strains	Antibiotics/ MICs[a] (μg/mL)	
	ETH	INH
WT	256	> 512
Δ*nudC*	16	1
ΔΔ*ethA1*	64	1
ΔΔ*ethA1*^{CMab}	16	1
ΔΔ*ethA1*^{CMtb}	32	1
ΔΔ*ethA1*^{CEV}	64	1
ΔΔ*ethA2*	32	1
ΔΔ*ethA2*^{CMab}	16	1
ΔΔ*ethA2*^{CMtb}	16	1
ΔΔ*ethA2*^{CEV}	32	1

[a] Broth microdilution method was used to determine the MICs. WT, wild-type *M. abscessus*; Δ*nudC*, *M. abscessus nudC* knockout strain; ΔΔ*ethA1*, *ethA1* (*MAB_0985*) and *nudC* double knockout strain; ΔΔ*ethA1*^{CMab}, ΔΔ*ethA1*^{CMtb}, ΔΔ*ethA1*^{CEV}, ΔΔ*ethA1* complemented with *ethA1*^{Mab}, *ethA*^{Mtb} or with the empty vector, respectively. ΔΔ*ethA2*, *ethA2* (*MAB_013*) and *nudC* double knockout strain; ΔΔ*ethA2*^{CMab}, ΔΔ*ethA2*^{CMtb}, ΔΔ*ethA2*^{CEV}, ΔΔ*ethA2* complemented with *ethA2*^{Mab}, *ethA*^{Mtb}, or with the empty vector, respectively.

Table S2 The susceptibility of *M. abscessus* strains overexpressing *ethA1*, *ethA2*, or *ethA3* in either wild-type or *nudC* deletion backgrounds to ETH and INH.

***M. abscessus* strains**	**Antibiotics/ MICs[a] (μg/mL)**	
	ETH	INH
Δ*nudC*^{OEV}	16	1
Δ*nudC*^{OethA1}	2	1
Δ*nudC*^{OethA2}	1	1
Δ*nudC*^{OethA3}	2	1
WT^{OEV}	256	512
WT^{OethA1}	128	512
WT^{OethA2}	256	512
WT^{OethA3}	256	512

[a] Broth microdilution method was used to determine the MICs. Δ*nudC*^{OEV}, Δ*nudC*^{OethA1}, Δ*nudC*^{OethA2}, Δ*nudC*^{OethA3}, *M. abscessus nudC* knockout strain containing an empty vector or the vector for overexpressing *ethA1*^{Mab}, *ethA2*^{Mab}, or *ethA3*^{Mab}, respectively. WT^{OEV}, WT^{OethA1}, WT^{OethA2}, WT^{OethA3}, wild-type *M. abscessus* containing an empty vector or the vector for overexpressing *ethA1*^{Mab}, *ethA2*^{Mab}, or *ethA3*^{Mab}, respectively.

Table S3 The sequences of all primers used in this study.

Primers	Sequences (5'-3')
MAB_0984c-crRNA1-F	atccggccgaccttctacttctaa
MAB_0984c-crRNA1-R	agctttagaagtagaaggtcggccggatct
MAB_0984c-crRNA2-F	attcctccaaagagtccgtactca
MAB_0984c-crRNA2-R	agcttgagtacggactctttggaggaatct
MAB_0984c-crRNA3-F	atggctcccaccgggccctcacta
MAB_0984c-crRNA3-R	agcttagtgagggcccggtgggagccatct
MAB_0985-crRNA1-F	atgtggtgctggaggcgcgcgagca
MAB_0985-crRNA1-R	agcttgctcgcgcgcctccagcaccacatct
MAB_0985-crRNA2-F	atggacatgttcacgctcggctaa
MAB_0985-crRNA2-R	agctttagccgagcgtgaacatgtccatct
MAB_0985-crRNA3-F	atggtgtgacagaaaaggtccgaa
MAB_0985-crRNA3-R	agctttcggaccttttctgtcacaccatct
MAB_0103-crRNA1-F	attcgccgtggaacgacagcaggaa
MAB_0103-crRNA1-R	agctttcctgctgtcgttccacggcgaatct
MAB_0103-crRNA2-F	attgctgcagtgggtactacgacta
MAB_0103-crRNA2-R	agcttagtcgtagtacccactgcagcaatct
MAB_0103-crRNA3-F	ataaaggcaccgtggtgcacccga
MAB_0103-crRNA3-R	agcttcgggtgcaccacggtgcctttatct
MAB_3967-crRNA1-F	atcggtatcccggtgtgcgttgcga
MAB_3967-crRNA1-R	agcttcgcaacgcacaccgggataccgatct
MAB_3967-crRNA2-F	attcctttgctccgtggaccggcaa
MAB_3967-crRNA2-R	agctttgccggtccacggagcaaaggaatct
MAB_3967-crRNA3-F	attcatggtgtgcacgggctacta
MAB_3967-crRNA3-R	agcttagtagcccgtgcacaccatgaatct
pMV-*MAB_0984c*-F	agacaattgcggatccgtgacaaaaccggctacgag
pMV-*MAB_0984c*-R	actacgtcgacatcgatctacgaattcgcacaaacccct
pMV-*MAB_0985*-F	agacaattgcggatccatgaccgaacatgtcgacattgt

Table S3 The sequences of all primers used in this study (continued).

Primers	Sequences (5'-3')
pMV-*MAB_0985*-R	actacgtcgacatcgatttacctgcctattgcaacggg
pMV-*MAB_0103*-F	agacaattgcggatccaatgtccgaacactttgacgtgc
pMV-*MAB_0103*-R	actacgtcgacatcgatctagctgttgacggccacc
pMV-*MAB_3967*-F	agacaattgcggatccatgaaccccgcagcagac
pMV-*MAB_3967*-R	actacgtcgacatcgattcacgataccgttgagtttcttgagaag
MAB_0985-F	gccgaacttcatcttcacgttc
MAB_0985-R	tctgcccttccttgtccatg
MAB_0103-F	tacaagggcaagaaggtggtc
MAB_0103-R	tgatgatggggttctcattggg
MAB_3967-F	tacacatcactgcccgtcac
MAB_3967-R	tccacggagcaaaggagaag

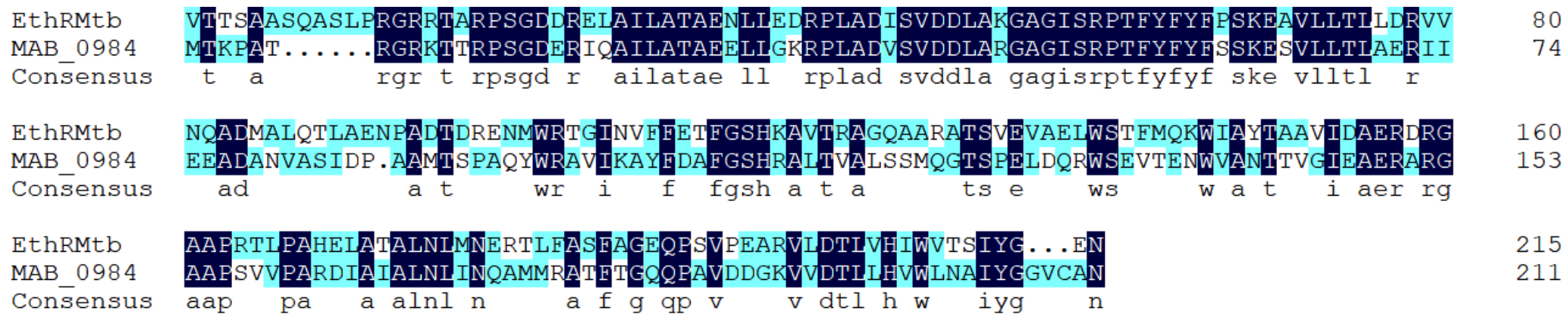

Fig. S1 Alignment of the MAB_0984 and *M. tuberculosis* EthR amino acid sequences.

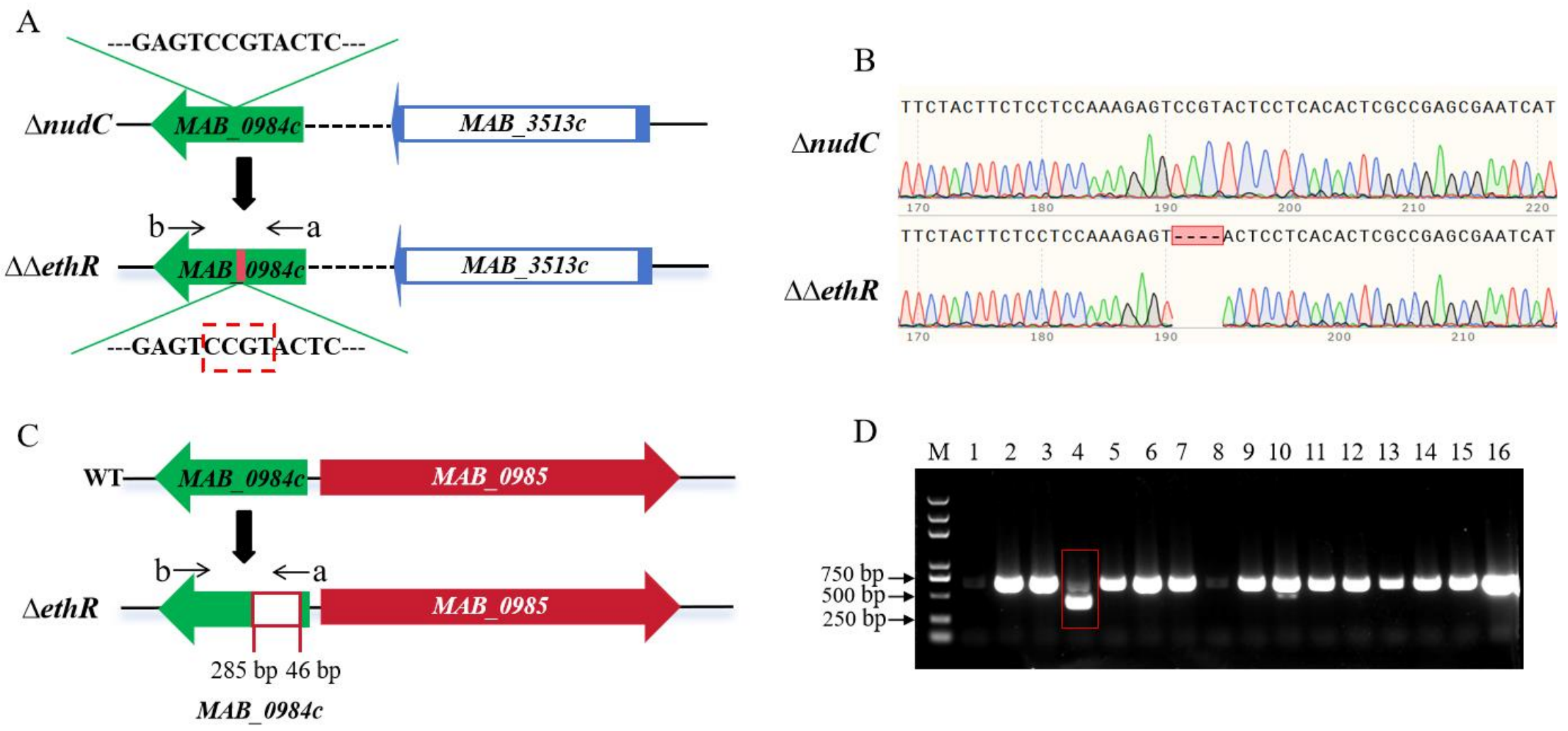

Fig. S2 Knockout of *ethR* (*MAB_0984c*) in either wild-type or *nudC* knockout strains. (A) Schematic representation of *ethR* and *nudC* double knockout strain (ΔΔ*ethR*). In the Δ*nudC* background, a 4-bp deletion (nucleotides 191-194) was introduced in *ethR*, generating a frameshift mutation near the 5' end of the gene. Δ*nudC*, *M. abscessus nudC* knockout strain; (**B**) Confirmation of the ΔΔ*ethR* knockout sequences by Sanger sequencing. (**C**) Schematic representation of *ethR* single knockout strain (Δ*ethR*). This mutant was constructed in the wild-type (WT) background via an in-frame deletion of nucleotides 46-285 of *ethR*. WT, wild-type *M. abscessus.* (**D**) Verification of the Δ*ethR* by PCR. Lane M, DNA ladder; lanes 1-15, Screening of potential Δ*ethR* mutants by PCR using primers a (pMV-*MAB_0984c*-F) and b (pMV-*MAB_0984c*-R), with lane 4 showing the product of the Δ*ethR*; lane 16, PCR product from WT using the same primers. The expected band sizes are ~650 bp for the WT allele and ~400 bp for the deletion allele, respectively.

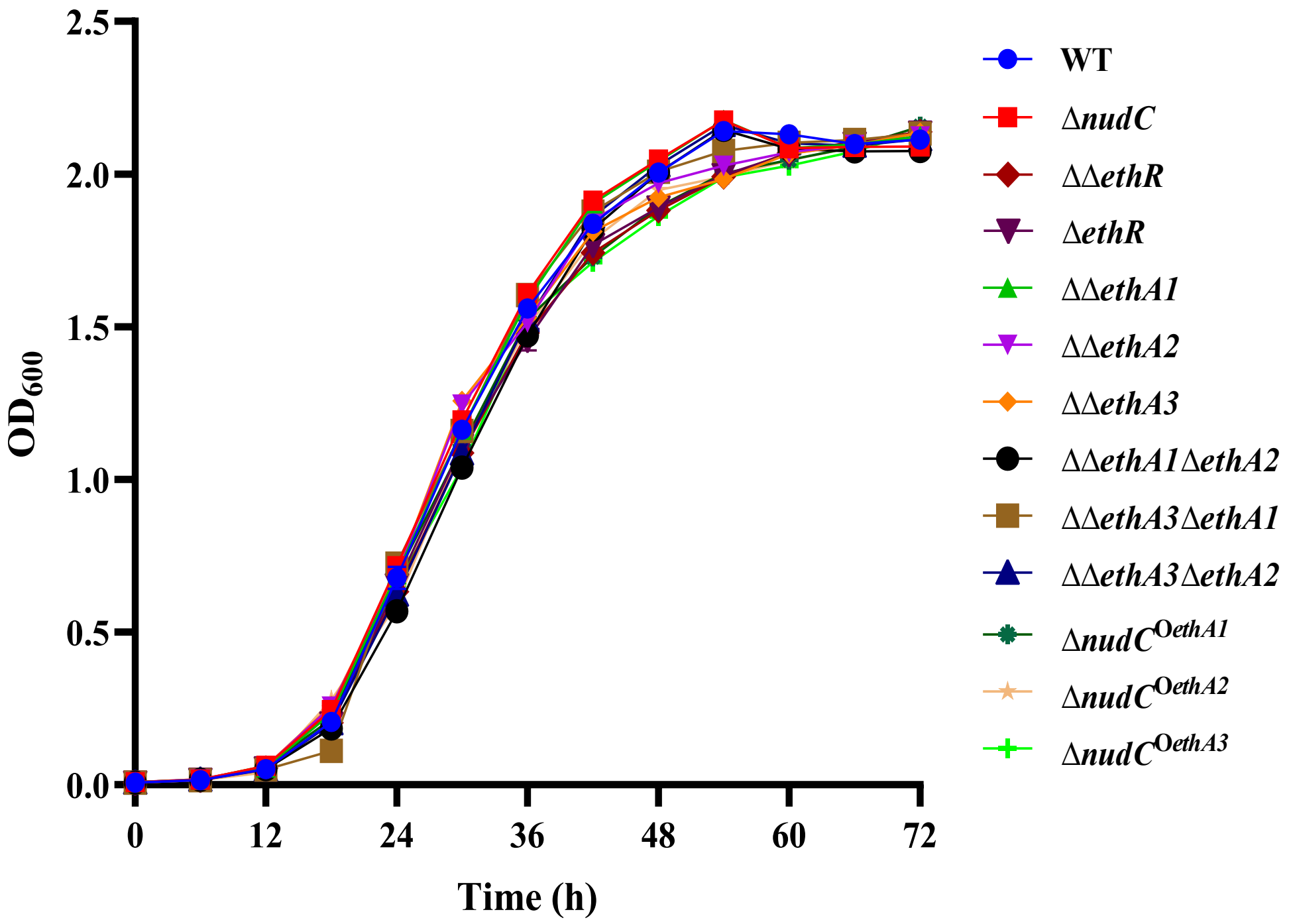

Fig. S3 Growth curves of various *M. abscessus* strains in 7H9 broth at 37°C. WT, wild-type *M. abscessus*; Δ*nudC*, *M. abscessus nudC* knockout strain; ΔΔ*ethR*, *ethR* (*MAB_0984c*) and *nudC* double knockout strain; ΔΔ*ethA1*, *ethA1* (*MAB_0985*) and *nudC* double knockout strain; ΔΔ*ethA2*, *ethA2* (*MAB_0103*) and *nudC* double knockout strain; ΔΔ*ethA3*, *ethA3* (*MAB_3967*) and *nudC* double knockout strain; ΔΔ*ethA1*Δ*ethA2*, *ethA1*, *ethA2* and *nudC* triple knockout strain; ΔΔ*ethA3*Δ*ethA1*, *ethA3*, *ethA1* and *nudC* triple knockout strain; ΔΔ*ethA3*Δ*ethA2*, *ethA3*, *ethA2*, and *nudC* triple knockout strain. Δ$nudC^{OethA1}$, Δ$nudC^{OethA2}$, Δ$nudC^{OethA3}$, *M. abscessus nudC* knockout strain containing an empty vector or the vector for overexpressing $ethA1^{Mab}$, $ethA2^{Mab}$, or $ethA3^{Mab}$, respectively.

A

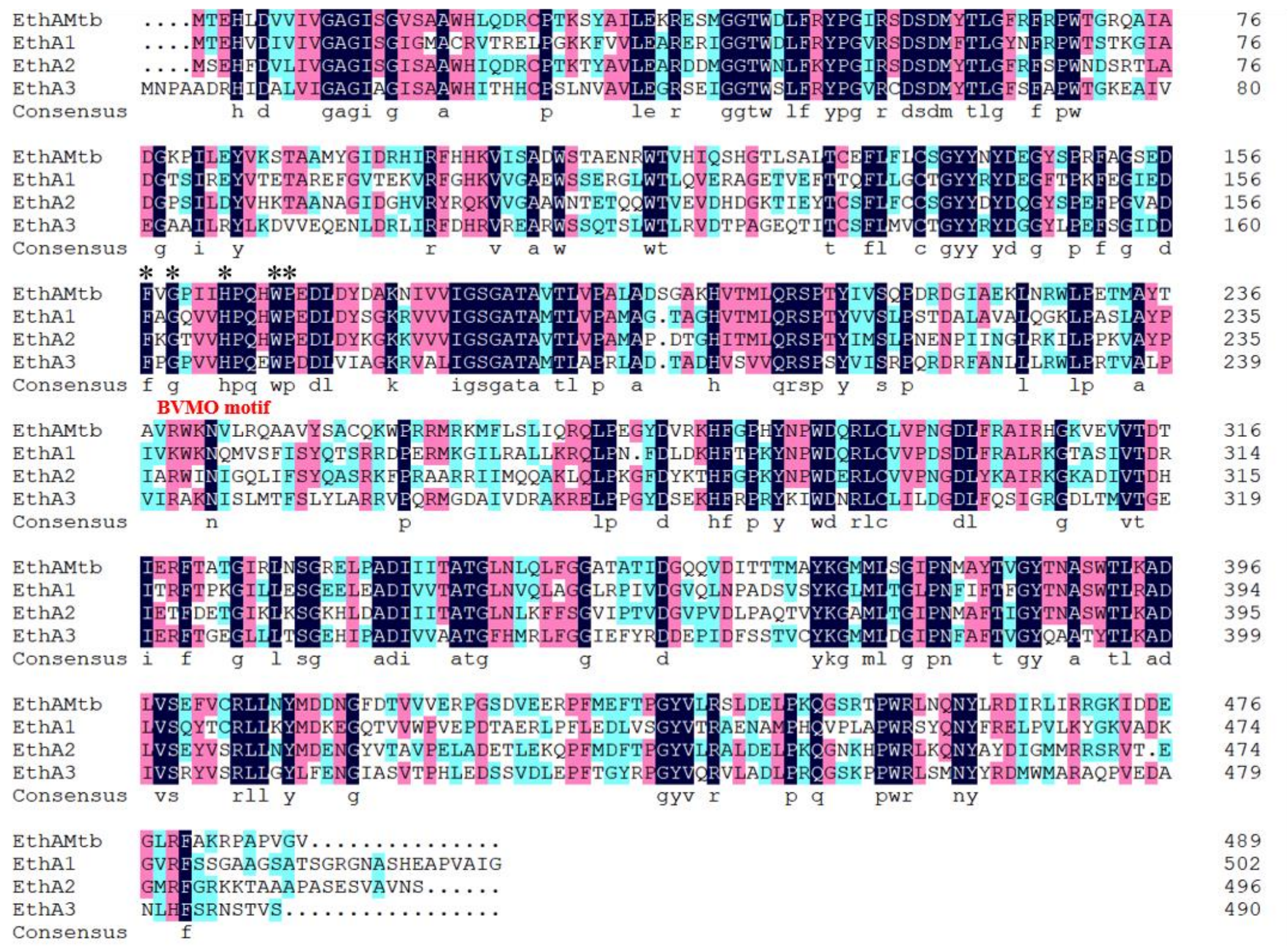

B

Protein	EthA^{Mtb}	EthA1	EthA2	EthA3
EthA^{Mtb}	100%	51.38	59.44%	47.46%
EthA1	51.38%	100%	51.09%	43.11%
EthA2	59.44%	51.09%	100%	45.11%
EthA3	47.46%	43.11%	45.11%	100%

Fig. S4 Alignment of the EthA amino acid sequences from *M. abscessus* and *M. tuberculosis*.

(**A**) The multisequence alignment of the EthA amino acid sequences from *M. abscessus* and *M. tuberculosis*. The asterisk (*) indicates the Baeyer-Villiger monooxygenase (BVMO) motif [FXGXXXHXXXW(P/D)]. (**B**) Results of pairwise amino acid sequence identity alignment of EthA1, EthA2, EthA3 of *M. abscessus* and EthA^{Mtb}.

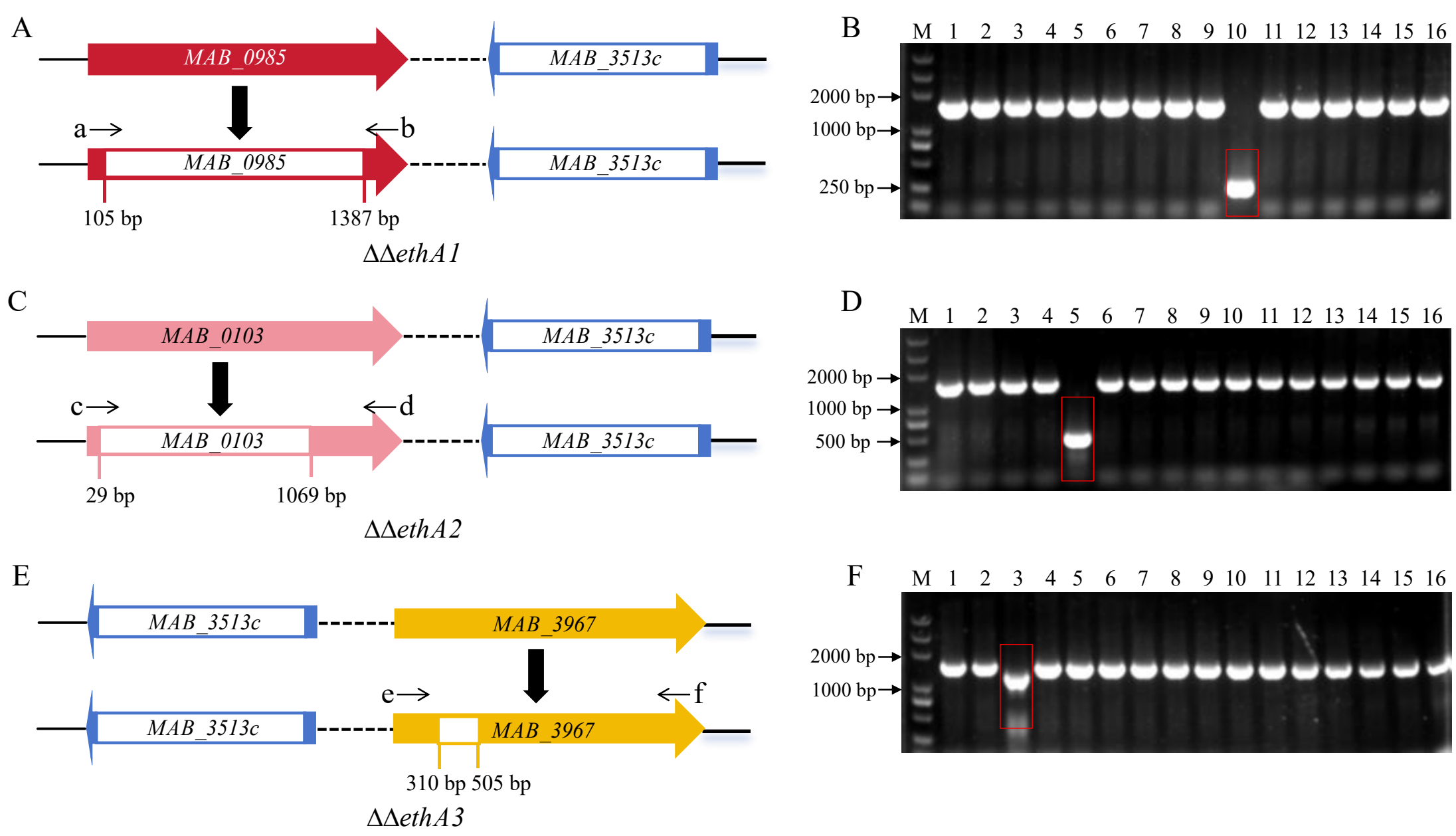

Fig. S5 Individual knockout of *ethA1* (*MAB_0985*), *ethA2* (*MAB_0103*), or *ethA3* (*MAB_3967*) in *nudC* knockout strain. (**A**) Schematic representation of *ethA1* and *nudC* double knockout strain (ΔΔ*ethA1*). The ΔΔ*ethA1* was constructed in the Δ*nudC* via a deletion of nucleotides 105-1387. (**B**) Verification of the ΔΔ*ethA1* by PCR. Lane M, DNA ladder; lanes 1-15, Screening of potential ΔΔ*ethA1* mutants by PCR using primers a (pMV-*MAB_0985*-F) and b (pMV-*MAB_0985*-R), with lane 10 showing the product of the Δ*ethA1*; lane 16, PCR product from WT using the same primers. The expected band sizes are ~1500 bp for the WT allele and ~250 bp for the deletion allele, respectively. (**C**) Schematic representation of *ethA2* and *nudC* double knockout strain (ΔΔ*ethA2*). The ΔΔ*ethA2* was constructed in the Δ*nudC* via an in-frame deletion of nucleotides 29-1069. (**D**) Verification of the ΔΔ*ethA2* by PCR. lanes 1-15, Screening of potential ΔΔ*ethA2* mutants by PCR using primers c (pMV-*MAB_0103*-F) and d (pMV-*MAB_0103*-R), with lane 5 showing the product of the Δ*ethA2*; lane 16, PCR product from WT using the same primers. The expected band sizes are ~1500 bp for the WT allele and ~500 bp for the deletion allele, respectively. (**E**) Schematic representation of *ethA3* and *nudC* double knockout strain (ΔΔ*ethA3*). The ΔΔ*ethA3* was constructed

in the Δ*nudC* via a deletion of nucleotides 310-505. (**F**) Verification of the ΔΔ*ethA3* by PCR. lanes 1-15, Screening of potential ΔΔ*ethA3* mutants by PCR using primers e (pMV-*MAB_3967*-F) and f (pMV-*MAB_3967*-R), with lane 3 showing the product of the Δ*ethA3*; lane 16, PCR product from WT using the same primers. The expected band sizes are ~1500 bp for the WT allele and ~1200 bp for the deletion allele, respectively.

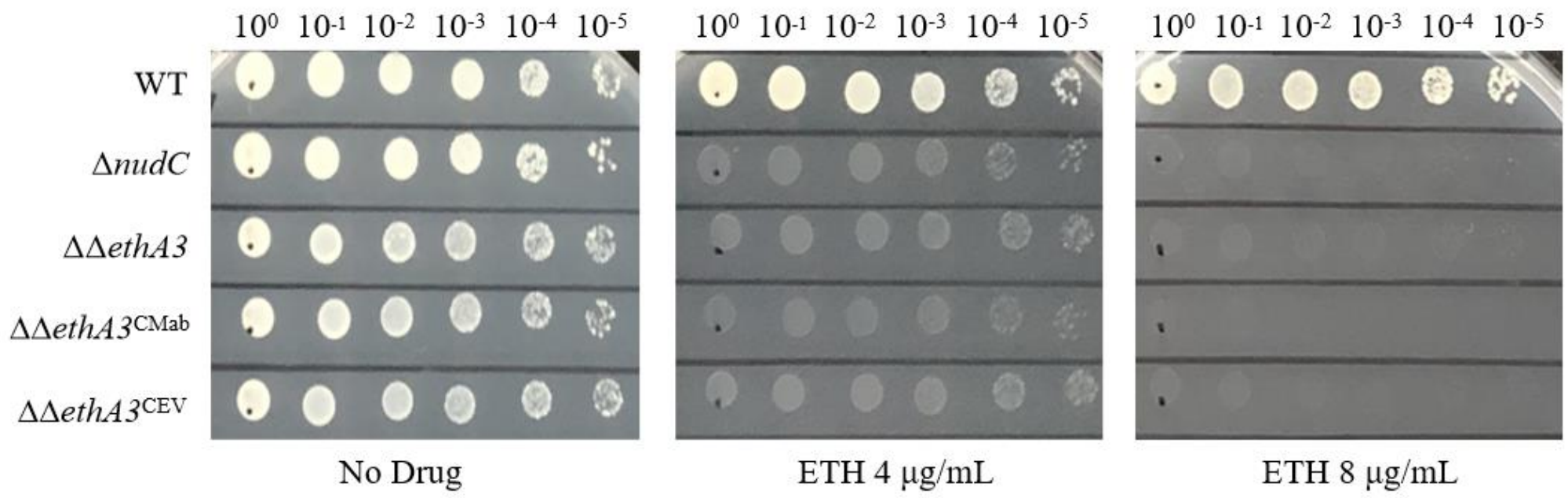

Fig. S6 Effect of *ethA3* deletion on ETH susceptibility in the Δ*nudC* background Drug susceptibility testing of *ethA3* and *nudC* double knockout strain to ETH. WT, wild-type *M. abscessus*; Δ*nudC*, *M. abscessus nudC* knockout strain; ΔΔ*ethA3*, *ethA3* and *nudC* double knockout strain; ΔΔ*ethA3*^{CMab} and ΔΔ*ethA3*^{CEV}, ΔΔ*ethA3* complemented with *ethA3*^{Mab} and the empty vector, respectively.

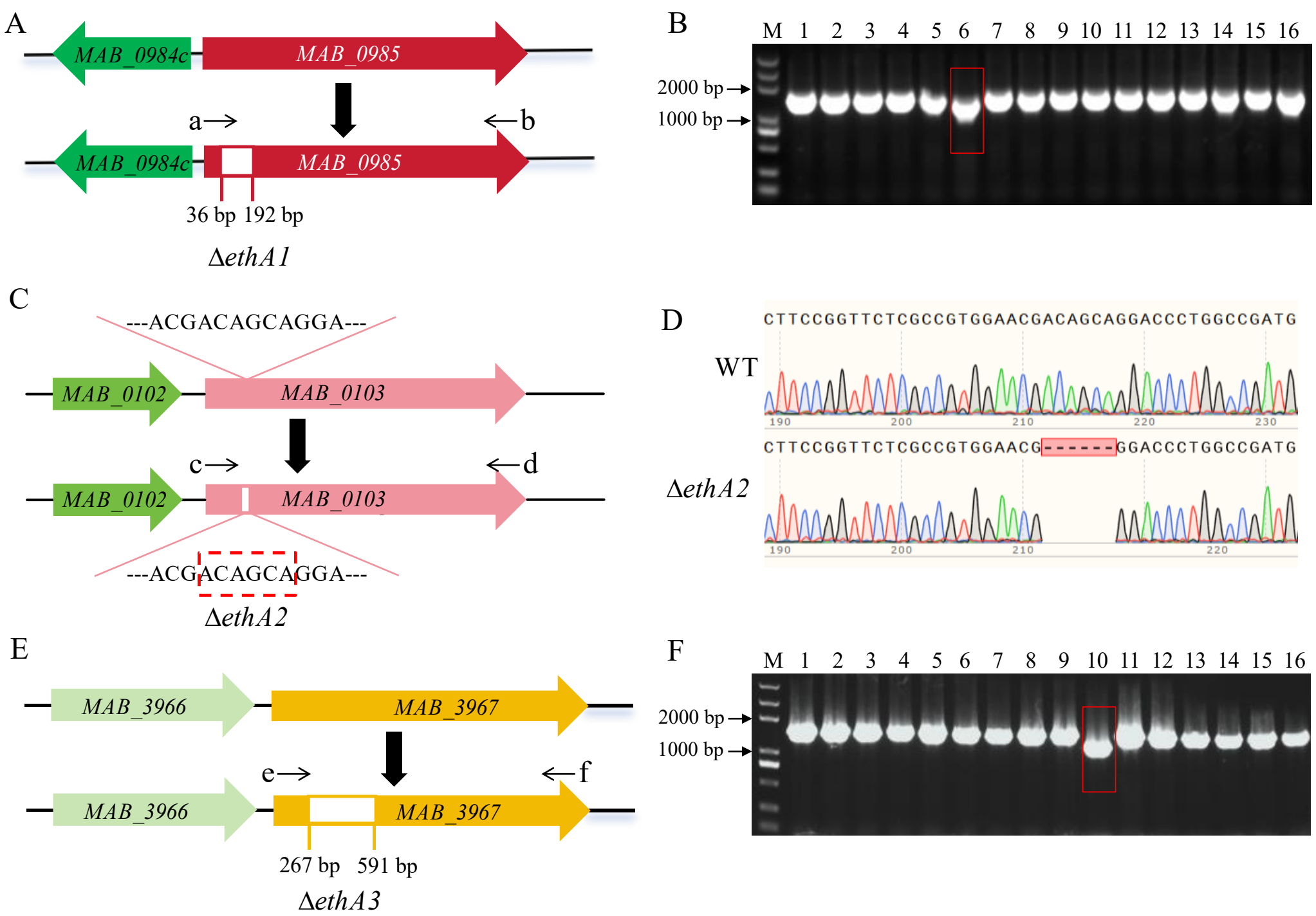

Fig. S7 Individual knockout of *ethA1* (*MAB_0985*), *ethA2* (*MAB_0103*), or *ethA3* (*MAB_3967*) in wild-type *M. abscessus*. (**A**) Schematic representation of *ethA1* knockout strain (Δ*ethA1*). The Δ*ethA1* was constructed in the WT via a deletion of nucleotides 36-192 in *ethA1*. (**B**) Verification of the Δ*ethA1* by PCR. Lane M, DNA ladder; lanes 1-15, Screening of potential Δ*ethA1* mutants by PCR using primers a (pMV-*MAB_0985*-F) and b (pMV-*MAB_0985*-R), with lane 6 showing the product of the Δ*ethA1*; lane 16, PCR product from WT using the same primers. The expected band sizes are ~1500 bp for the WT allele and ~1350 bp for the deletion allele, respectively. (**C**) Schematic representation of *ethA2* knockout strain (Δ*ethA2*). A 6-bp deletion was introduced to generate an in-frame deletion near the 5' end of *ethA2*. (**D**) Confirmation of the Δ*ethA2* knockout sequence by Sanger sequencing. (**E**) Schematic representation of *ethA3* knockout strain (Δ*ethA3*). The Δ*ethA3* was constructed in the WT via a deletion of nucleotides 267-591 in *ethA3*. (**F**) Verification of the Δ*ethA3* by PCR. lanes 1-15, Screening of potential Δ*ethA3* mutants by PCR using primers e (pMV-*MAB_3967*-F) and f (pMV-*MAB_3967*-R), with lane 10 showing the product of the Δ*ethA3*; lane 16, PCR product from WT using the same primers. The expected band sizes are ~1500 bp for the

WT allele and ~1150 bp for the deletion allele, respectively.

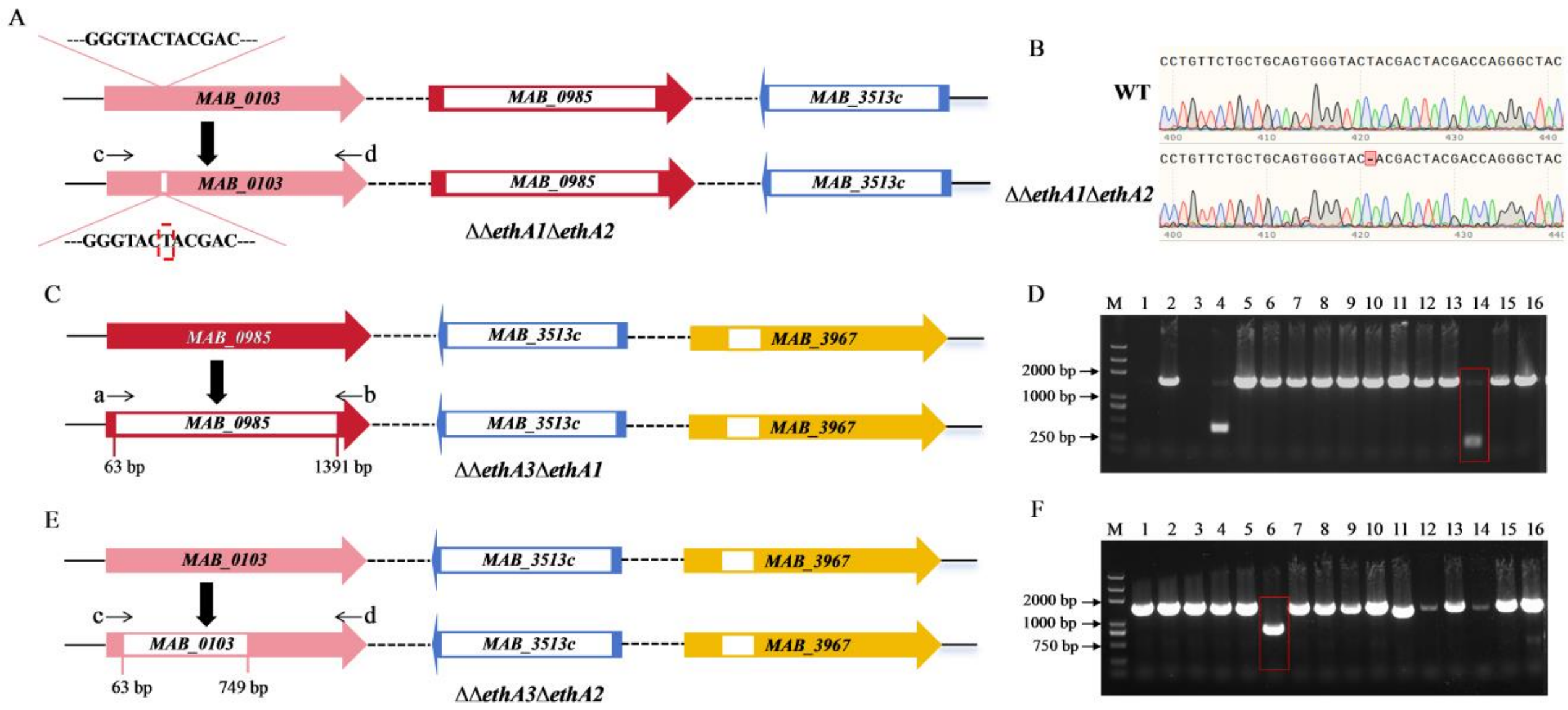

Fig. S8 Pairwise deletion of *ethA1* (*MAB_0985*), *ethA2* (*MAB_0103*), and *ethA3* (*MAB_3967*) in *nudC* knockout strain. (**A**) Schematic representation of *ethA1*, *ethA2*, and *nudC* triple knockout strain (ΔΔ*ethA1*Δ*ethA2*). The ΔΔ*ethA1*Δ*ethA2* was constructed in the ΔΔ*ethA1* via a deletion of nucleotides 421 in *ethA2*. (**B**) Confirmation of the ΔΔ*ethA1*Δ*ethA2* knockout sequence by Sanger sequencing. (**C**) Schematic representation of *ethA1*, *ethA3*, and *nudC* triple knockout strain (ΔΔ*ethA3*Δ*ethA1*). The ΔΔ*ethA3*Δ*ethA1* was constructed in the ΔΔ*ethA3* via an in-frame deletion of nucleotides 63-1391 in *ethA1*. (**D**) Verification of the ΔΔ*ethA3*Δ*ethA1* by PCR. lanes 1-15, Screening of potential ΔΔ*ethA3*Δ*ethA1* mutants by PCR using primers a (pMV-*MAB_0985*-F) and b (pMV-*MAB_0985*-R), with lane 14 showing the product of the ΔΔ*ethA3*Δ*ethA1*; lane 16, PCR product from WT using the same primers. The expected band sizes are ~1500 bp for the WT allele and ~200 bp for the deletion allele, respectively. (**E**) Schematic representation of *ethA2*, *ethA3*, and *nudC* triple knockout strain (ΔΔ*ethA3*Δ*ethA2*). The ΔΔ*ethA3*Δ*ethA2* was constructed in the ΔΔ*ethA3* via a deletion of nucleotides 63-749 in *ethA2*. (**F**) Verification of the ΔΔ*ethA3*Δ*ethA2* by

PCR. lanes 1-15, Screening of potential Δ*ΔethA3ΔethA2* mutants by PCR using primers c (pMV-*MAB_0103*-F) and d (pMV-*MAB_0103*-R), with lane 6 showing the product of the Δ*ΔethA3ΔethA2*; lane 16, PCR product from WT using the same primers. The expected band sizes are ~1500 bp for the WT allele and ~800 bp for the deletion allele, respectively.

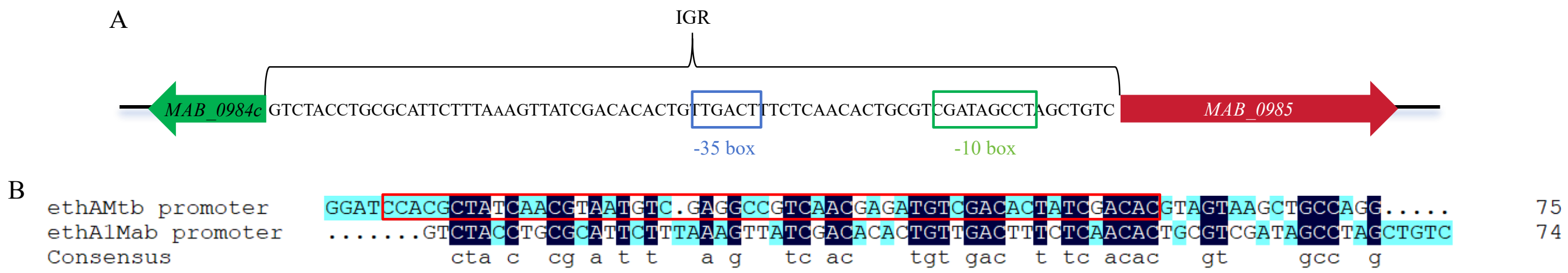

Fig. S9 Analysis of the intergenic sequence (IGR) between *ethR* (*MAB_0984c*) and *ethA1* (*MAB_0985*). (**A**) The nucleotide sequence of the *M. abscessus ethA1* (*MAB_0985*) promoter region. The putative -10 and -35 boxes are marked with green and blue boxes, respectively. (**B**) Sequence alignment of the *M. abscessus ethA1* promoter region with that of *M. tuberculosis ethA*. The EthR^{Mtb} binding site is marked with red box.

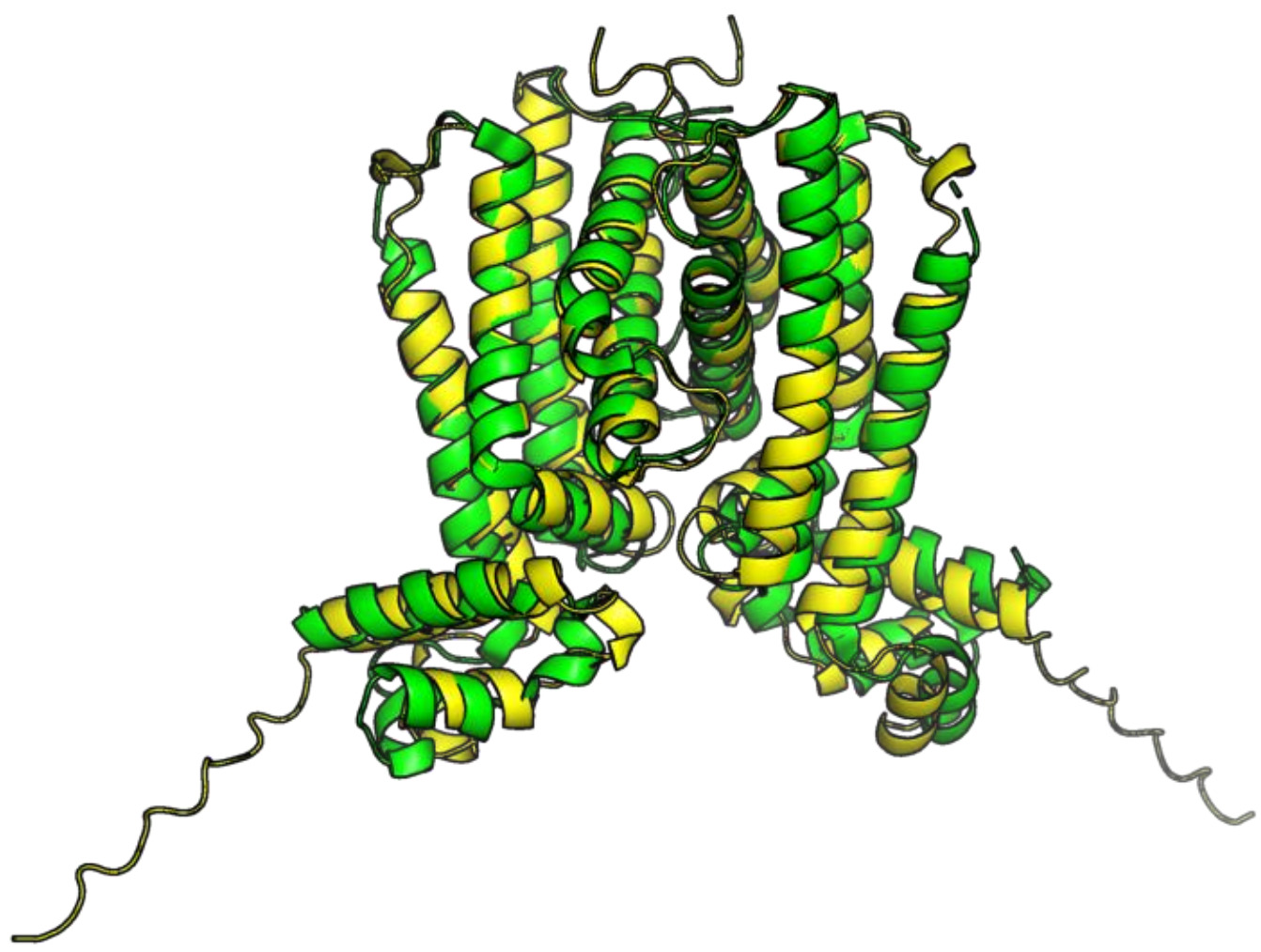

Fig. S10 Structural alignment of the predicted dimeric EthR structures from *M. abscessus* and *M. tuberculosis*. Yellow, EthR^{Mab} dimeric structure predicted by AlphaFold3; Green, EthR^{Mtb} dimeric structure (PDB code: 5NZ1).

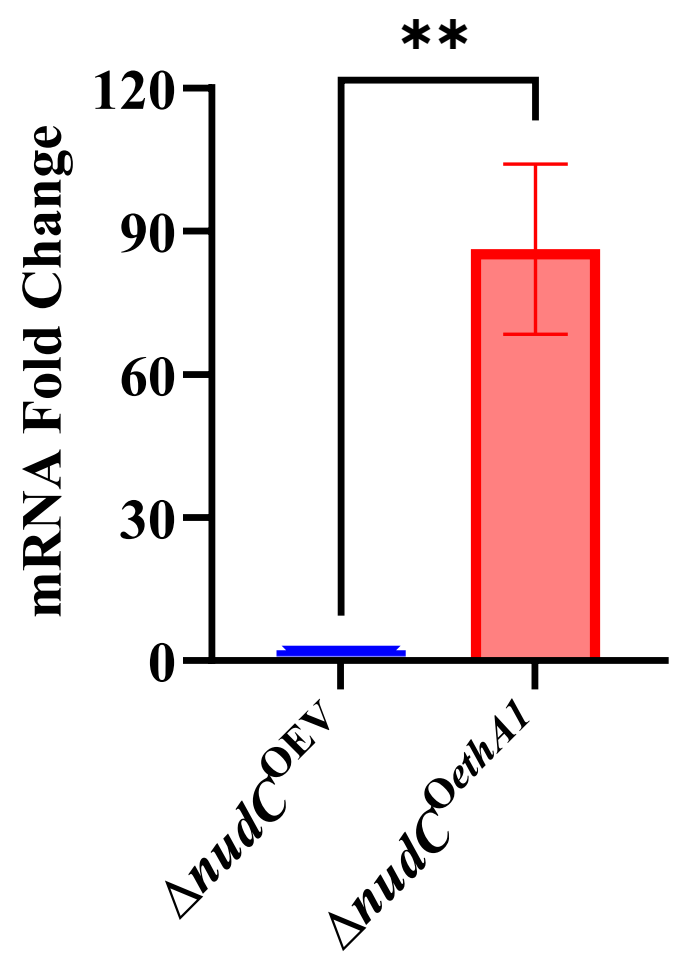

Fig. S11 Gene expression levels of *ethA1* in the overexpression strain. Δ*nudC*^{OEV} and Δ*nudC*^{OethA1}, *M. abscessus nudC* knockout strain containing an empty vector or the vector for overexpressing *ethA1*^{Mab}, respectively; $^{**}P < 0.01$. Data are means ± SD of three biological replicates.
